# Multiscale Spatial Fusion Feature‐Driven Characterization of Gastric Cancer Invasive Margins: A Multicenter Cohort Study for Preoperative Accurate Differentiation Between T4a and T4b Subtypes

**DOI:** 10.1002/advs.76455

**Published:** 2026-07-20

**Authors:** Guoliang Zheng, Xiaomiao Chai, Peng Jin, Xin Xin, Yingjie Li, Chun Yang, Jia Wei, Zishuo Yan, Jingyu Zhang, Qianning Zhao, Yingchen Han, Ning Zhang, Fuze Li, Bo Qiao, Huan Wang, Huachuan Zheng, Yan Li, Xin Zhang, Yan Zhao, Wenjun Mao, Jing Zhang

**Affiliations:** ^1^ Department of Gastric Surgery Cancer Hospital of Dalian University of Technology Liaoning Cancer Hospital & Institute Shenyang Liaoning China; ^2^ School of Pharmacy China Medical University Shenyang Liaoning China; ^3^ Department of Gastric Surgery National Clinical Research Center for Cancer Tianjin Key Laboratory of Digestive Cancer Tianjin Medical University Cancer Institute and Hospital Tianjin Clinical Research Center for Cancer Tianjin China; ^4^ Shenyang Mental Health Center Shenyang Liaoning China; ^5^ Department of Radiology Hunnan Central Hospital Shenyang Liaoning China; ^6^ Department of Medical Oncology The First Hospital of China Medical University Shenyang Liaoning China; ^7^ School of Management Hebei University Baoding Hebei China; ^8^ Department of Pathology Cancer Hospital of Dalian University of Technology Liaoning Cancer Hospital & Institute Shenyang Liaoning China; ^9^ Department of Radiotherapy Cancer Hospital of China Medical University Liaoning Cancer Hospital & Institute Cancer Hospital of Dalian University of Technology Shenyang Liaoning China; ^10^ Center of Translational Medicine and Department of Gastroenterology The First Affiliated Hospital of Jinzhou Medical University Jinzhou Liaoning China; ^11^ Department of Laboratory Medicine Institute of Laboratory Medicine Sichuan Provincial People's Hospital School of Medicine University of Electronic Science and Technology of China Chengdu Sichuan China; ^12^ Department of Nuclear Medicine Shengjing Hospital of China Medical University Shenyang Liaoning China; ^13^ Department of Thoracic Surgery The Affiliated Wuxi People's Hospital of Nanjing Medical University Wuxi Medical Center Nanjing Medical University Wuxi Jiangsu China

**Keywords:** computed tomography, deep learning, gastric cancer, multicenter study, semantic segmentation, T staging, visual transformer

## Abstract

Accurate preoperative differentiation of gastric cancer T4a/b stages is crucial for surgical planning and prognosis. However, conventional CT assessments often yield suboptimal staging accuracy due to visual limitations and inadequate peritumoral microinvasion quantification. This study developed a multi‐scale spatial feature fusion model based on extended regions of interest (eROI) for precise preoperative T4a/b differentiation. We proposed the GAVR model with a three‐tier architecture: a Boundary‐Augmented U‐Net for eROI generation incorporating the peritumoral microenvironment; parallel pathways extracting conventional radiomics, 2D, and 3D deep learning features; and a Vision Transformer for global attention‐weighted fusion and discriminative representation learning. The model was validated across a multicenter cohort of 1804 patients, including internal, external, and prospective sets. A blinded reader study involving 16 radiologists evaluated its clinical utility. GAVR demonstrated exceptional generalizability, achieving AUCs of 0.987 and 0.979 in two independent external sets and 0.987 prospectively. Ablation studies confirmed the necessity of multi‐scale features. GAVR assistance significantly improved radiologists' diagnostic accuracy (0.609 to 0.795) and reduced reading time by 60%. By deeply fusing multi‐scale spatial features, GAVR characterizes structural heterogeneity in complex gastric cancer invasive margins and mitigates overfitting. It demonstrates clear translational value as an embeddable decision‐support tool for multidisciplinary gastric cancer management.

## Graphical abstract

This study develops a multi‐dimensional vision Transformer‐based model, GAVR, to accurately distinguish gastric cancer T4a/b stages preoperatively. Validated across multi‐center cohorts, it achieves excellent performance and significantly improves radiologists’ diagnostic accuracy, offering a promising tool for clinical decision‐making.

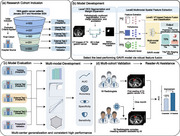

## 1 Introduction

Gastric cancer is an important public health problem worldwide, and its incidence and mortality have significant regional differences. According to GLOBOCAN2022 data, about 71.4% of new cases and 70.1% of deaths occur in Asia, especially in East Asia [1]. Among them, China bears the heaviest disease burden, with the number of cases and deaths of gastric cancer ranking among the highest in the world [2], suggesting that the situation of prevention and treatment of gastric cancer in East Asia, especially in China, is still grim, and more effective diagnosis and treatment strategies are urgently needed to improve the prognosis of patients.

In the staging system of gastric cancer, the depth of tumor invasion (T stage) is one of the key factors to determine the treatment plan and evaluate the prognosis [3]. According to the depth of invasion, it is divided into T1 to T4 stages. Stage T4 was divided into T4a and T4b subtypes. T4a indicated that the tumor had invaded the serosa layer but not adjacent tissues or organs. T4b means that the tumor has invaded the adjacent organs or structures, such as the pancreas, liver, colon, etc [4]. Although the difference in anatomical staging between the two is relatively subtle, its clinical significance is completely different. Patients with T4a stage usually only undergo partial/total gastrectomy with D2 lymph node dissection [5]. However, once the disease progresses to stage T4b, the surgical strategy often needs to be adjusted to more complex treatment schemes such as combined organ resection [6, 7]. Previous studies have shown that the postoperative 5 year survival rate of patients with T4b gastric cancer is only about 10%–32%, and the prognosis is significantly poor [8]. If the T4b stage is not accurately identified before surgery, but it is found during surgery that the tumor has invaded adjacent organs or tissues, it is often forced to temporarily change the surgical plan, which not only increases the risk of perioperative complications but also may affect the trust of patients and their families in the treatment process [9]. Therefore, in addition to the evaluation of lymph nodes and distant metastasis, it is particularly important to determine whether the tumor has invaded adjacent organs when making treatment decisions [10, 11]. In conclusion, accurate identification of T4a and T4b before surgery is of great clinical value for rational surgical planning, evaluation of tumor resectability, and realization of individualized treatment.

At present, preoperative imaging evaluation (mainly enhanced CT) is the cornerstone examination method for T staging and overall preoperative staging of gastric cancer [12]. However, CT still has obvious limitations in distinguishing T4 substaging. Radiologists mainly rely on indirect signs such as blurred perigastric fat space, disappearance of the fat plane between the tumor and adjacent organs, and interruption of the organ interface to make judgments [13]. As a result, the imaging interpretation of T4a and T4b is highly dependent on subjective experience and difficult to diagnose [14]. Therefore, preoperative CT staging errors are common, especially the overdiagnosis or missed diagnosis of T4b. Previous studies have reported that only about 42%–66% of patients with T4b evaluated by imaging are confirmed to be true T4b lesions by postoperative pathology [15], suggesting that the extent of tumor invasion in a considerable proportion of patients is overestimated before surgery, which may affect the reasonable choice of treatment strategy. In addition to enhanced CT, endoscopic ultrasonography (EUS) is also commonly used for preoperative evaluation of gastric cancer [16]. However, its ability to discriminate locally advanced lesions (T3‐T4), especially T4a and T4b, is limited by factors such as penetration depth, inflammatory reaction, and operator experience [17].

In recent years, the rapid development of artificial intelligence (AI) has provided a new solution to the problem of imaging staging. Radiomics and deep learning methods based on medical images have shown great potential in the diagnosis, staging and prognosis prediction of gastric cancer [18, 19]. However, most of the existing studies focus on the prediction of overall T staging, while the intelligent models specifically for T4 substaging (T4a and T4b) have not yet emerged [20]. In addition, previous AI studies generally have problems such as small sample size, single‐center design, lack of external validation, and insufficient generalization ability of models [21]. Some models only focus on the tumor itself and do not fully incorporate key information such as tumor‐surrounding tissues and adjacent organ invasion, thus limiting their ability to discriminate T4 substaging [22].

GAVR model proposed in this study aims at the key clinical challenge that it is difficult to quantitatively evaluate the microinvasion of the tumor. Through the multi‐dimensional Vit architecture, the GAVR model systematically integrates traditional imaging features, high‐order abstract features, 3D spatial features, and important clinical information, and overcomes the human visual limitation in the identification of gastric cancer T4a/T4b. In particular, the clinical bottleneck of quantitative assessment of peritumoral microinvasion was explored, and the possibility of AI to redefine the tumor boundary identification criteria was explored. The proposed model has been rigorously validated on multi‐center and large sample datasets, showing high accuracy and good generalization ability.

## 2 Materials and Methods

### 2.1 Patient Recruitment Process and Data Set Composition

**FIGURE 1:**
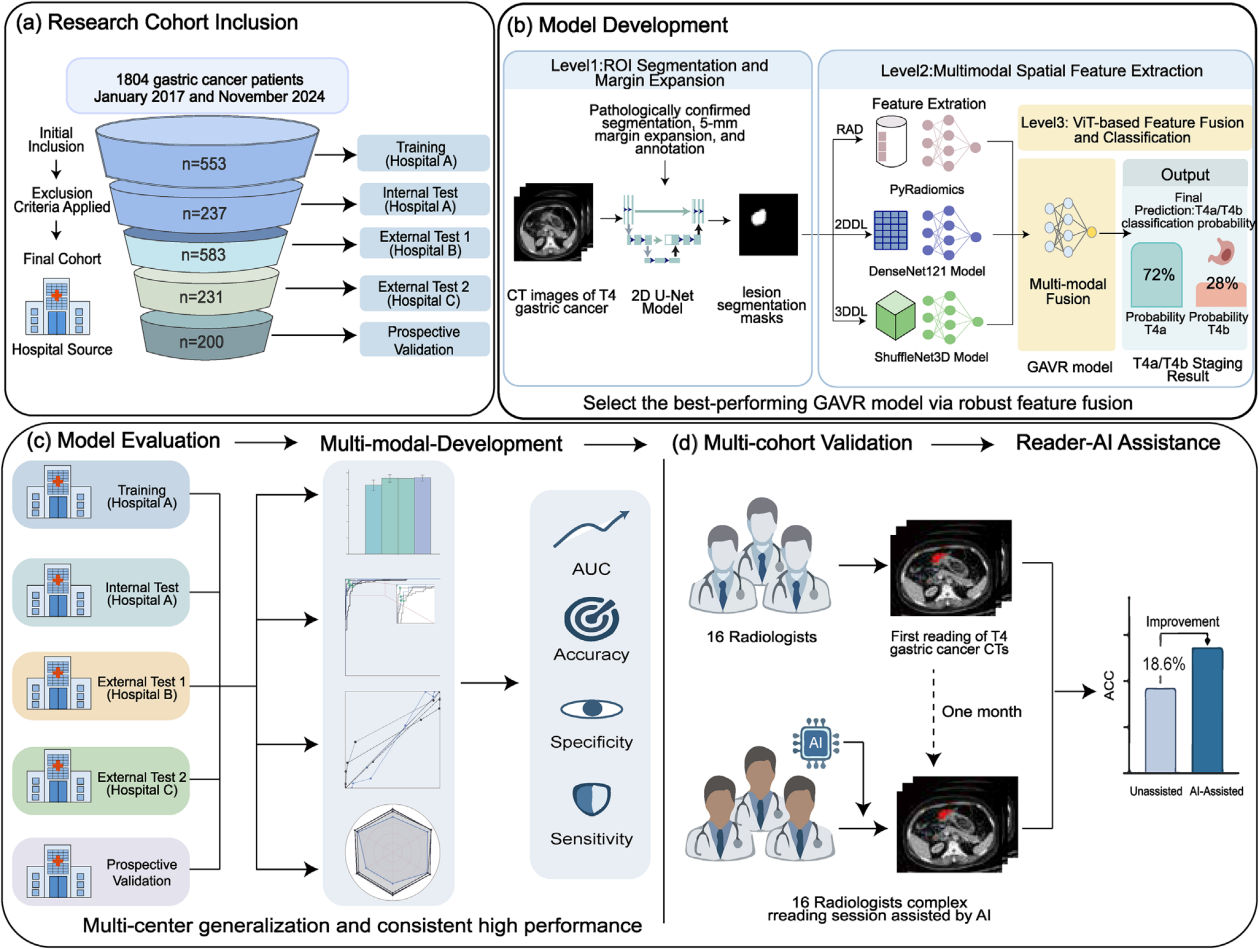
Study Design and Workflow. (a) Study cohort construction. From January 2017 to November 2024, a total of 1804 consecutive patients with gastric cancer were enrolled in this study. According to the prespecified inclusion and exclusion criteria, the following data sets were finally formed: the training set (*n* = 553) and the internal test set (*n* = 237) from Hospital A (Liaoning Cancer Hospital); External test set 1 (*n* = 583) from hospital B (Shengjing Hospital of China Medical University); External test set 2 (*n* = 231) from hospital C (Tianjin Medical University Cancer Institute and Hospital); And the prospective validation set from hospital A (*n* = 200); And (b)GAVR model construction process. Firstly, BAE‐U‐Net was used for automatic segmentation of eROI, and then classical radiomics features, two‐dimensional deep learning features, and three‐dimensional deep learning features were extracted from eROI images, and multi‐dimensional features were fused by ViT to construct the GAVR model. (c) Model performance evaluation. The receiver operating characteristic (ROC) curve, calibration curve, and radar chart were used to comprehensively evaluate the discriminant ability and prediction consistency of the model. (d) AI‐assisted clinical decision‐making evaluation. Sixteen radiologists independently evaluated 40 cases from 200 prospective cases in two rounds. With the assistance of the GAVR model, the overall diagnostic accuracy increased by about 18.6%, indicating the potential value of the model in clinical decision‐making.

The data of this study were obtained from Liaoning Cancer Hospital (Hospital A), Shengjing Hospital of China Medical University (Hospital B), and Tianjin Medical University Cancer Institute and Hospital (Hospital C). The study adhered to the ethical principles of the Declaration of Helsinki and was approved by the ethics committee of each center. The CT imaging data and clinical information of 1804 patients with gastric cancer were included. According to the data source, they were divided into a training set, an internal test set, an external test set 1, an external test set 2, and a prospective validation set for the construction and validation of the model (Figure 1). The inclusion criteria included: (1) gastric cancer in stage T4a or T4b confirmed by pathology (Note: cases with mesocolon invasion leading to partial colon resection were also included as T4b group); (2) CT scan was completed within one month before surgery; (3) complete imaging data. Exclusion criteria included: (1) poor image quality; (2) previous history of gastric surgery, radiotherapy, or chemotherapy; (3) incomplete imaging or clinical data.

The T stage of all patients was determined according to the American Joint Committee on Cancer (AJCC) eighth edition TNM staging system. Baseline clinical information was extracted from the electronic medical record system. Demographic characteristics (age, sex), tumor anatomical characteristics (tumor location, tumor size), histological characteristics (Lauren classification, degree of differentiation), molecular biological characteristics (HER2 status, MMR status), and preoperative serum tumor marker levels (CEA, AFP, CA19‐9, CA72‐4, and CA125) were collected (Table 1).

**TABLE 1:** Characteristics of enrolled gastric cancer patients in the development, internal, and external datasets (mean ± standard deviation or n (%)).

charecteristics	Training set (*n* = 553)			Internal test set (*n* = 237)			External test set1 (*n* = 583)			External test set2 (*n* = 231)			Prospective set (*n* = 200)		
	T4a^a^(*n* = 265)	T4b^a^(*n* = 288)	*p*‐value	T4a(*n* = 125)	T4b(n = 112)	*p*‐value	T4a(*n* = 511)	T4b(*n* = 72)	*p*‐value	T4a(*n* = 100)	T4b(*n* = 131)	*p*‐value	T4a(*n* = 105)	T4b(*n* = 95)	*p*‐value
Age (years)	63.42 ± 9.68	64.12 ± 9.96	0.503	62.73 ± 11.29	64.07 ± 11.37	0.221	63.35 ± 9.51	64.62 ± 10.62	0.169	64.50 ± 9.48	66.59 ± 8.83	0.086	63.10 ± 7.83	64.42 ± 9.77	0.351
Sex(%)			1			0.565			0.044			1			1
female	81 (30.57)	88 (30.56)		37 (29.60)	38 (33.93)		163 (31.90)	14 (19.44)		33 (33.00)	43 (32.82)		26 (24.76)	24 (25.26)	
Male	184 (69.43)	200 (69.44)		88 (70.40)	74 (66.07)		348 (68.10)	58 (80.56)		67 (67.00)	88 (67.18)		79 (75.24)	71 (74.74)	
Tumor location (%)^b^			0.002			0.739			0.340			0.033			0.357
L	164 (61.9)	152 (52.8)		64 (51.2)	52 (46.4)		289 (56.56)	42 (58.33)		54 (54.00)	86 (65.6)		50 (47.6)	52 (54.7)	
M	72 (27.2)	73 (25.3)		38 (30.4)	36 (32.1)		140 (27.40)	23 (31.94)		10 (10.00)	18 (13.7)		37 (35.2)	33 (34.7)	
U	29 (10.9)	63 (21.9)		23 (18.4)	24 (21.4)		82 (16.05)	7 (9.72)		36 (36.00)	27 (20.6)		18 (17.1)	10 (10.5)	
Tumor size(cm)	5.05 ± 2.48	4.92 ± 2.59	0.453	5.59 ± 2.51	5.15 ± 2.41	0.233	4.31 ± 2.31	5.67 ± 2.91	<0.001	4.19 ± 2.31	5.73 ± 2.66	<0.001	4.58 ± 2.76	4.26 ± 2.36	0.419
Lauren (%)^c^			<0.001			0.003			0.068			0.493			0.81
intestinal	70 (26.4)	118 (34.0)		34 (27.2)	54 (48.2)		184 (36.01)	20 (27.78)		45 (45.00)	49 (37.40)		41 (39.0)	33 (37.4)	
mixed	107 (40.4)	69 (24.0)		46 (36.8)	26 (23.2)		139 (27.20)	29 (40.28)		22 (22.00)	32 (24.43)		27 (28.4)	27 (28.4)	
diffuse	88 (33.2)	101 (35.1)		45 (36.00)	32 (28.6)		188 (36.79)	23 (31.94)		33 (33.00)	50 (38.17)		37 (35.2)	35 (36.8)	
Differentiation status(%)			0.87			0.718			0.029			0.707			0.523
well	13 (4.9)	17 (5.9)		6 (4.8)	4 (3.6)		72 (14.09)	5 (6.94)		6 (6.00)	5 (3.82)		7 (6.7)	3 (3.2)	
moderate	53 (20.0)	58 (20.1)		29 (23.2)	31 (27.7)		138 (27.01)	13 (18.06)		29 (29.00)	36 (27.48)		20 (19.0)	19 (20.0)	
poor	199 (75.1)	213 (74.0)		90 (72.0)	77 (68.8)		301 (58.90)	54 (75.00)		65 (65.00)	90 (68.70)		78 (74.3)	73 (76.8)	
her2(%)^d^			0.709			0.915			0.149			0.809			0.182
negative	232 (87.55)	248 (86.11)		110 (88.00)	100 (89.29)		423 (82.78)	65 (90.28)		86 (86.00)	110 (83.97)		101 (96.19)	86 (90.53)	
positive	33 (12.45)	40 (13.89)		15 (12.00)	12 (10.71)		88 (17.22)	7 (9.72)		14 (14.00)	21 (16.03)		4 (3.81)	9 (9.47)	
MMR (%)^d^			1			1			<0.001			0.716			0.006
pMMR	255 (96.23)	277 (96.18)		122 (97.60)	110 (98.21)		501 (98.04)	63 (87.50)		96 (96.00)	128 (97.71)		31 (29.52)	12 (12.63)	
dMMR	10 (3.77)	11 (3.82)		3 (2.40)	2 (1.79)		10 (1.96)	9 (12.50)		4 (4.00)	3 (2.29)		74 (70.48)	83 (87.37)	
CEA, ng/mL^e^	25.94 ± 14.42	24.85 ± 13.99	0.601	26.08 ± 13.22	23.50 ± 12.51	0.199	14.67 ± 8.49	15.79 ± 9.99	0.639	14.98 ± 9.56	14.84 ± 10.08	0.674	23.50 ± 16.55	17.39 ± 16.01	0.004
AFP, ng/mL^e^	9.41 ± 5.79	9.99 ± 5.08	0.062	9.38 ± 5.99	9.61 ± 4.49	0.373	8.06 ± 2.55	8.64 ± 5.17	0.138	8.79 ± 4.68	8.97 ± 5.60	0.884	18.39 ± 8.29	16.79 ± 8.34	0.098
CA199,U/mL^e^ ^)^	49.43 ± 28.86	47.44 ± 25.90	0.583	48.05 ± 29.41	47.74 ± 21.85	0.628	38.76 ± 15.66	36.65 ± 23.55	0.705	39.65 ± 24.76	29.49 ± 25.66	0.002	45.15 ± 34.76	46.79 ± 32.89	0.913
CA724,U/mL^e^ ^)^	28.63 ± 17.01	28.75 ± 16.01	0.8	29.64 ± 16.75	29.22 ± 16.21	0.845	17.60 ± 10.54	18.40 ± 15.48	0.393	16.68 ± 11.95	15.88 ± 13.83	0.185	11.21 ± 16.90	11.63 ± 17.97	0.765
CA125,U/mL^e^ ^)^	36.77 ± 23.06	31.47 ± 16.91	0.001	35.42 ± 22.85	32.38 ± 17.17	0.287	26.65 ± 10.52	20.61 ± 16.68	<0.001	22.46 ± 14.29	17.10 ± 14.80	0.005	25.69 ± 25.96	23.87 ± 25.67	0.288

^a^ T4a and T4b are TNM stages of the tumor.

^b^ Tumor anatomical location was divided into lower (L), middle (M), and upper (U) stomach.

^c^ Lauren classification was divided into intestinal type, diffuse type, and mixed type, and the degree of differentiation was divided into well differentiated, moderately differentiated, and poorly differentiated.

^d^ Molecular features included HER2 status (negative/positive) and MMR status (pMMR/dMMR).

^e^ Serum tumor markers included PD‐L1 (%), CEA (ng/mL), AFP (ng/mL), CA19‐9, CA72‐4 and CA125 (U/mL).

### 2.2 Overall Architecture of GAVR Model and CT Image Preprocessing Scheme

The core innovation of the GAVR model is to construct a modular three‐level architecture, which integrates multi‐dimensional features based on eROI and enhances the complementarity among these features through the self‐attention mechanism of Vit, so as to achieve accurate assessment between the tumor and the surrounding organs. The first‐level architecture implements automatic segmentation of the tumor eROI through the “Boundary‐Aware Extended U‐Net” (BAE‐U‐Net) model to realize the marginal expansion and automatic segmentation of the primary tumor. The second‐level architecture realizes multi‐dimensional spatial feature extraction through three parallel sub‐models. The third‐level architecture realizes the integration and learning of multi‐dimensional spatial features through Vit and makes classification decisions. All enrolled patients underwent preoperative abdominal computed tomography (CT). Given that data from multiple centers may differ with respect to CT scan device models, scan parameters, and image acquisition protocols, detailed device and parameter specifications are listed in Table S1 and Section S1 to ensure comparability of the data and the generalization of the model.

#### 2.2.1 First‐Level Architecture—Semantic Segmentation Through BAE‐U‐Net

Firstly, the classical UNet model is trained for automatic image segmentation. Training used data from the manual annotation segmentation mask (mask), the work marked by three experienced abdominal imaging diagnostic radiologists in the Deepwise medical imaging platform (https://keyan.deepwise.com/login/). The labeling process was as follows: First, the cases were equally assigned to the three physicians, and the two‐dimensional slices containing the tumors in their CT images were accurately delineated layer by layer; Subsequently, all the labeling results were independently reviewed by the fourth physician. If any disagreement was found, the relevant physicians would discuss and reach a consensus. If consensus could not be reached, a third senior radiologist made final adjudication. All segmentation masks were manually reviewed for validation and included in the model training. The input of the model was the normalized 2D CT image slice, and the output was the corresponding binary tumor segmentation mask. The binary cross‐entropy loss function was used in the training process, and the Adam optimizer was used to update the parameters. The model continued to iterate on the training set until the loss function converged, and then the optimal model was saved for subsequent prediction tasks. The detailed model parameter configuration and training process are shown in Figure 3a and Section S2.

**FIGURE 2:**
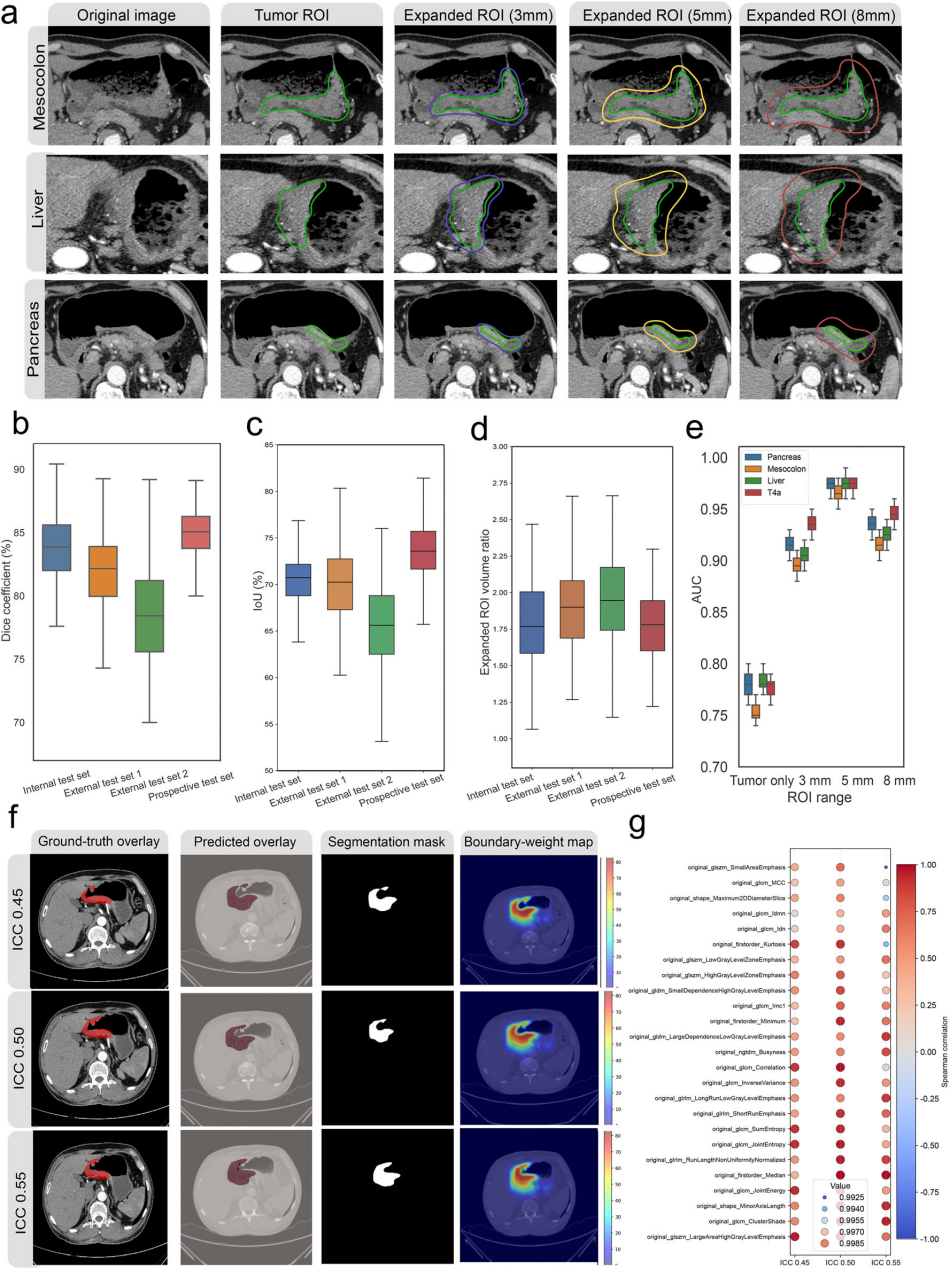
Results of systematic evaluation of the semantic segmentation performance of the BAE‐U‐Net model. (a) Schematic diagram of the construction process of eROI based on the BAE‐U‐Net model. From left to right, the original CT images of T4 gastric cancer, the traditional tumor ROI (green outline), and the eROI with different extension ranges (3, 5, and 8 mm) were shown. (b) Box plot of Dice similarity coefficient of raw segmentation results in four independent test cohorts; (c) box plot of intersection ratio (IoU) of original segmentation results in each test cohort; (d) Box plot of eROI volume expansion ratio in each test cohort; (e) Box plot of adjacent organ invasion subtype classification performance (AUC) under different peritumoral extension ranges (3, 5, and 8 mm); (f) Visualization comparison of manually labeled ROI and eROI under different segmentation thresholds (0.45, 0.50, 0.55). From left to right, they are: artificial segmentation mask overlay map, eROI overlay map, binary segmentation mask, and pixel‐level loss weight map. (g) Dot heatmap of Spearman correlation coefficient between radiomics features and the consensus vector under three segmentation thresholds (0.45, 0.50, 0.55). The color represents the magnitude of the correlation coefficient, and the size of the points reflects the absolute value of the correlation, which is used to evaluate the robustness of the feature representation under segmentation perturbation.

In order to minimize the potential impact of image segmentation uncertainty on the feature extraction of the secondary architecture, this study proposes a “bounding‐aware Extended U‐Net” model based on the classical U‐Net model: An isotropic expansion operation based on voxel spacing was implemented on the original segmentation mask to construct an expanded region of interest (eROI) to incorporate more peritumor microenvironment regions (Figure 2a). The core advantage of this strategy is that even if there are local uncertainties in the original segmentation results (such as boundary shift or small missed segmentation), the expanded region covered by eROI can still robustly retain the anatomical interface information between the tumor and adjacent organs, so as to effectively alleviate the conductive interference of segmentation error on the multi‐dimensional feature extraction of secondary architecture. In order to determine the optimal extent of eROI expansion, we designed three expansion ranges (3, 5, and 8 mm). The volume and ratio of the original ROI to eROI were calculated according to the voxel spacing, which was used as the key index to optimize the eROI boundary expansion width. Dice Similarity Coefficient (DSC) was used to quantify the spatial consistency of eROI masks at different thresholds. For masks with inconsistent spatial resolution, the nearest neighbor interpolation method was used for resampling and alignment. Finally, the discriminative robustness under the perturbation of segmentation uncertainty was systematically evaluated by comparing the different eROI configurations in the T4 sub‐classification task (Figure 2e). In addition, in order to simulate the unstable segmentation results of eROI, three levels of judgment thresholds of 0.45, 0.50, and 0.55 were set for BAE‐U‐Net, respectively, corresponding to three eROI segmentation masks with different boundaries. Radiomics features were extracted independently under each threshold condition (Figure 2f). Heatmaps of Spearman correlation coefficients between radiomics features and the three threshold consensus vectors were used to evaluate the stability of features under segmentation perturbation conditions (Figure 2g).

#### 2.2.2 Secondary Architecture—Three Independent Submodels Achieve eROI Multi‐Dimensional Feature Extraction

The second‐level architecture of the GAVR model consists of three independent sub‐models. Each sub‐model takes eROI images as input, extracts multi‐dimensional image features, and connects to the three‐level fusion architecture in parallel. Specifically, channel 1 extracted radiomics features based on PyRadiomics, and all computational parameters were set according to its official documentation specifications to generate multi‐dimensional quantitative image representations including morphological features, first‐order statistical features, and texture features. Channel 2 extracted two‐dimensional deep learning features based on the DenseNet121 model (model selection was based on comparative experimental results; see Table S2 for details), and obtained 1024‐dimensional feature vectors corresponding to each ROI image block from the penultimate layer of the network. Considering that it is difficult for a single slice to comprehensively reflect the spatial information of tumors, this study adopted a strategy of including all tumor slices for feature aggregation to construct a complete two‐dimensional deep learning feature representation. Channel 3 extracts 3D DL features based on ShuffleNet3D. Bounding box clipping was performed on the tumor region based on the segmentation mask and uniformly resampled into fixed‐size volume data (64 × 64 × 64) to construct a standardized 3D input. Subsequently, 567‐dimensional feature vectors were extracted by the global average pooling layer of ShuffleNet3D (model selection based on comparative experimental results, see Table S3 for details) to characterize the 3D spatial structure information of tumors, thereby making up for the limitations of 2D features in spatial modeling.

After the completion of multi‐dimensional feature extraction, in order to further evaluate the distribution characteristics of different modal features and their role in the model, a multi‐dimensional feature analysis framework was constructed. Specifically, the t‐SNE method was used to reduce and visualize the different modal features to evaluate their distribution patterns in the low‐dimensional space. Based on the multi‐dimensional feature fusion model completed by training, the importance of input features was quantified by the SHAP method. In addition, by calculating the response strength of different modal features in different stages and types of organ invasion, the feature activation map was constructed to describe the expression patterns of different modal features in different clinical situations.

#### 2.2.3 Three‐Level Architecture: Multi‐Dimensional Feature Fusion Based on ViT Framework

The third‐level cognitive architecture of the GAVR model is responsible for receiving three types of features from the previous first‐level architecture: interpretable machine learning features, two‐dimensional deep learning features, and three‐dimensional deep learning features. ViT is used to realize the dynamic weight allocation of the spatial relationship between tumors and surrounding organs through the self‐attention mechanism. The ViT framework consists of three independent modal coding branches, each of which contains a fully connected layer (for linear transformation), ReLU activation function, and Dropout layer (with a drop rate of 0.3) to complete the embedded feature representation within each mode and suppress overfitting. Subsequently, the features of the three channels are encoded as embedding vectors in their corresponding channels, and all the modal features are transformed into 64‐dimensional representations by linear mapping. After normalization, all embedding vectors are sent to the ViT architecture as input tokens for multi‐dimensional spatial relationship collaborative modeling. In the Transformer encoder, each modal token first interacts through the multi‐head self‐attention (MHSA) mechanism to capture the global dependence between different modes. Subsequently, the nonlinear high‐dimensional feature transformation is realized by the feed‐forward network (FFN). Residual connection and layer normalization were introduced into each coding layer to enhance the stability and generalization ability of deep network training. The final output is processed by the 128‐dimensional full connection layer, ReLU activation function, and Dropout layer (dropout rate 0.5), and then input to the MLP classification head, and the Softmax function is used to complete the two classification tasks of T4a and T4b. In order to verify the effectiveness of the Transformer fusion framework, this study carried out ablation experiments (Figure S1 in the Appendix) to show the performance comparison of Transformer fusion, feature fusion, and result fusion on four independent test sets.

**FIGURE 3:**
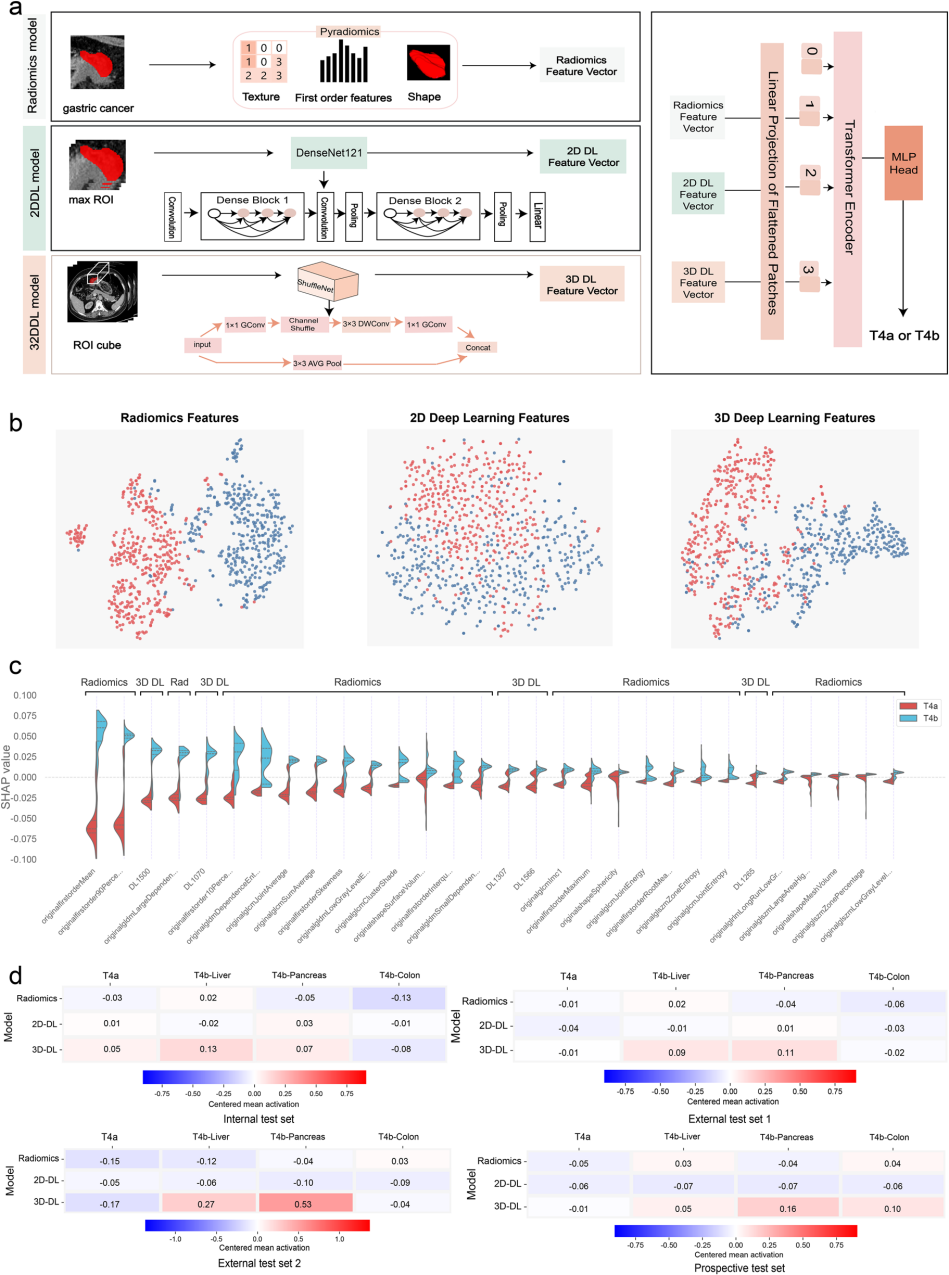
Multi‐dimensional feature extraction, fusion strategy, and model interpretability analysis. (a) Schematic diagram of the high‐dimensional modeling framework, including radiomics, 2D and 3D DL feature extraction, and VT‐based fusion process; (b) t‐SNE dimensionality reduction visualization of different modal features to demonstrate its discriminative ability in distinguishing T4a and T4b; (c) importance distribution of top 30 key features based on SHAP analysis; (d) The average activation intensity heatmaps of different modal features in the four validation sets under different organ invasion patterns at T4a and T4b were used to analyze the response differences of the models in different invasion scenarios. The heatmap values represent the centered mean activation, with positive values indicating higher than the overall average and negative values indicating lower than the average.

The cross‐entropy loss function was used to train the model. Adam was used as the optimizer (initial learning rate was 0.001, weight decay coefficient was 1 × 10^−^^4^), and the StepLR strategy was used to decay the learning rate to 0.1 times of the original value every 10 epochs. The data set was divided into a training set and a validation set according to the ratio of 7:3. During the training process, the changing trend of the loss function and classification accuracy was monitored in real time, and the model parameters with the best performance on the validation set were saved. The final model was comprehensively evaluated on the independent test set, and the output included key evaluation indicators such as classification accuracy, confusion matrix, and area under the ROC curve (AUC). In order to further improve the discrimination robustness of the model in complex clinical scenarios, a stratified sampling strategy for different organ invasion types (such as pancreas, liver, colon, and multiple organ invasion) in T4b was introduced in the training stage of this study. By guiding the model to focus on the differential representation signals of different invaded regions in the feature space, the network is encouraged to learn more discriminative cross‐regional feature expression patterns, thereby enhancing its ability to identify complex T4b lesions. The detailed model parameter configuration and training process are shown in Figure 3a and Section S3.

### 2.3 GAVR Model Performance Evaluation

GAVR model adopts a modular architecture design, and radiomics features, two‐dimensional deep learning features and three‐dimensional deep learning features are used as independent input channels to access the ViT fusion backbone. In order to systematically evaluate the collaborative modeling ability of multi‐dimensional heterogeneous features of the ViT fusion framework and quantify the relative contribution of each dimensional feature to the final prediction decision, a series of structured ablation experiments were conducted. The experiments covered an internal test set, two independent external validation cohorts, and a prospective multicenter cohort to comprehensively compare the full GAVR model with six types of ablation variants. The performance of three single‐channel ablation variants (radiomics only, 2D deep learning only, and 3D deep learning only) and three dual‐channel ablation variants (radiomics +2D deep learning, radiomics +3D deep learning, and 2D deep learning +3D deep learning) on a number of key performance indicators were compared. At the same time, the class discrimination ability of the model was visualized by the prediction probability distribution, and the reliability and Calibration of the prediction probability were comprehensively evaluated combined with the Calibration Curve and Brier score. The discrimination ability, prediction stability, and cross‐population generalization performance of the model were described in a multi‐dimensional and systematic way.

In addition, given the complex anatomy of the abdominal cavity and many adjacent organs, the characteristics of local invasion of T4b gastric cancer are highly dependent on the type of organ involved (such as colon, pancreas or liver). In order to evaluate the robustness of the GAVR model in discriminating different adjacent organ invasion patterns, this study further conducted a subgroup analysis based on the involved organs to systematically investigate its diagnostic performance in the three subgroups of colon invasion, pancreas invasion, and liver invasion.

### 2.4 Reader Study

To systematically evaluate the application value of the GAVR model in real clinical scenarios, this study designed an image interpretation experiment that simulated a standardized clinical workflow. A total of 16 qualified radiologists were enrolled (see Table S4 for details). All the radiologists were told that the images to be interpreted were from patients with pathologically or clinically confirmed stage T4 gastric cancer, but the specific distribution ratio of stage T4a and T4b cases was not informed. The imaging data were obtained from the prospective cohort of this study, from which enhanced CT images of 40 patients with T4 gastric cancer were randomly selected (20 cases of T4a stage and 20 cases of T4b stage). In the first interpretation stage, physicians independently completed the T4 subtype classification (T4a or T4b) according to the American Joint Committee on Cancer (AJCC) eighth edition TNM staging criteria for T staging on CT images. To minimize recall bias, the interval between the two interpretations was at least 30 days. Then, the same group of doctors performed a second interpretation on the same 40 images with complete randomization without providing a diagnosis, but only presenting the visual heat map and probability output of the GAVR model.

### 2.5 Statistical Analysis

In this study, PyTorch 1.1.0 was used to build the deep learning framework, and CUDA 11.3.1 and cuDNN 8.2.1 were used for low‐level acceleration optimization to improve the efficiency of model training and inference. Radiomics feature extraction was completed by PyRadiomics 3.0.1. The implementation of support vector machine (SVM) and other classical machine learning algorithms relies on Scikit‐learn 1.0. All statistical modeling and data analysis were completed using Statsmodels 0.13.2 in a Python 3.7.12 environment.

## 3 Results

### 3.1 Clinical Characteristics of the Study Cohort

Table 1 systematically shows the distribution of baseline characteristics of 1804 patients with gastric cancer from five independent datasets, including training set (*n* = 533, 30.7%), internal validation set (*n* = 237, 13.1%), external validation set 1 (*n* = 583, 32.3%), external validation set 2 (*n* = 231, 12.8%), and prospective validation set (*n* = 200, 11.1%).

### 3.2 Systematic Evaluation of Semantic Segmentation Performance of BAE‐U‐Net Model

As shown in Figure 2a, on the basis of the classical Region of Interest (ROI), the BAE‐U‐Net framework constructs expanded ROI (eROI) with different expanding ranges to incorporate the peri‐tumor microenvironment information. On this basis, the reliability of segmentation results was first evaluated in this study. In four independent test cohorts, rois generated by classical U‐Net and manually labeled rois showed high consistency, with an average Dice similarity coefficient of 0.82 ± 0.04 (Table 2, Figure 2b) and an average Intersection over Union (IoU) of 0.69 ± 0.05 (Table 2, Figure 2c), indicating that the segmentation model can stably and accurately identify tumor regions. Furthermore, to quantify the spatial variation of eROI relative to the original ROI, the volume expansion characteristics in different test cohorts were analyzed. The results showed that the eROI volume was enlarged by about 1.82–1.95 times on average compared with the original ROI (Figure 2d), while still maintaining a high spatial overlap (Dice = 0.68–0.73). The results showed that eROI could effectively expand the boundary area of the tumor while preserving the main spatial structure of the tumor.

**TABLE 2:** Evaluation of regional agreement between classic ROI and eROI (5 mm).

Test cohort	N (cases)	Traditional Dice coefficient (mean ± SD, %)	Traditional IoU (mean ± SD, %)	Expanded ROI volume ratio (mean ± SD)	Overlap between traditional and expanded ROI (Dice, mean ± SD, %)
Internal test set	231	84.0 ± 3.0	70.5 ± 2.9	1.82 ± 0.28	71.2 ± 7.5
External test set 1	278	82.0 ± 3.0	69.5 ± 4.2	1.88 ± 0.30	70.3 ± 8.0
External test set 2	231	79.0 ± 4.0	65.3 ± 4.8	1.95 ± 0.34	67.8 ± 8.6
Prospective test set	200	85.0 ± 2.0	73.9 ± 3.1	1.78 ± 0.26	72.5 ± 7.2

On this basis, we further evaluated the effects of different scaling strategies on the performance of downstream tasks. In the external validation cohort, the PyRadiomics (Rad) platform was used to extract the radiomics features of eROI obtained from the classic U‐Net segmentation ROI and three tumor margin expansion strategies (3, 5, 8 mm), respectively, and the discrimination efficacy (AUC) for tumor invasiveness classification was compared. The results showed that the 5 mm expansion strategy achieved the best performance in all invasive subtypes, with an average AUC of 0.98 (Figure 2e). This result suggests that moderate peritumoral extension can help to capture key information about the tumor‐stroma interface region, thereby enhancing the ability to characterize the invasive phenotype.

To further analyze the segmentation results from a qualitative perspective (Figure 2f), this study investigated the performance of BAE‐U‐Net at different segmentation thresholds (0.45, 0.50, and 0.55). The results showed that the boundary morphology of the eROI overlay map (Figure 2f) showed quantifiable differences with the change of threshold. However, under segmentation perturbation, the extracted radiomics features showed high robustness—the Intraclass Correlation Coefficient (ICC) of the vast majority of features were ≥ 0.90 (Figure 2g). It was confirmed that the eROI strategy could effectively suppress the conductive effect of segmentation error on the secondary analysis step (i.e., radiomics feature extraction and modeling). More comprehensive segmentation performance evaluation results (including Dice/IoU distribution, typical visualization cases, and error distribution analysis based on boundary distance) are provided in Figures S2 and S3 in the Supporting Appendix.

### 3.3 Visual Analysis of Multi‐Dimensional Feature Extraction Results

In the process of multi‐dimensional feature extraction, the three types of image features show good stability and usability. As shown in Figure 3a, the 2D and 3D deep learning channels extract high‐dimensional feature representations based on the pre‐trained network, respectively. The above features together constitute the input of the multi‐dimensional fusion model to provide complementary tumor representation information. The results of t‐SNE dimensionality reduction (Figure 3b) showed that the radiomics features and 3D deep learning features showed a relatively clear category distribution in the low‐dimensional space, and the boundaries between categories were relatively clear. In contrast, the clustering structure of 2D deep learning features was relatively loose, and the class discrimination was relatively low, suggesting that there were differences in the discriminative ability of different dimensional features. As shown in Figure 3c, the SHAP analysis shows the top 30 contributing features in the model. Different modal features all play an important role in the decision‐making of the model, and features from radiomics and 3D deep learning channels account for a relatively high proportion of high‐contributing features, which further reflects the complementarity between multi‐dimensional features. Feature activation analysis (Figure 3d) further revealed the differential responses of different dimensional features in characterizing tumor invasion patterns. In general, 3D deep learning features showed high activation strength in different organ invasions of T4b, especially pancreas and liver invasion, suggesting its advantages in capturing the spatial relationship between tumor and adjacent organs. The radiomics features showed a relatively stable and discriminative response pattern in different stages, especially in liver and colon invasion with high activation levels. In contrast, the activation intensity of 2D DL features in each category was relatively low and the variation range was limited, suggesting that the ability of 2D DL features to characterize complex spatial invasion patterns was relatively weak. The above results indicate that different dimensional features have different information expression abilities in different invasion situations, and there is a significant complementary relationship among the three, which provides an important basis for subsequent multi‐dimensional feature fusion.

### 3.4 Full GAVR Model and Six Types of Ablation Variant Performance Evaluation

The six types of ablation variants include three types of single‐channel ablation variants (Radiomics, 2DDL, and 3DDL) and three types of dual‐channel ablation variants (Radiomics + 2DDL, Radiomics + 3DDL, and 2DDL + 3DDL). The complete GAVR model is better than the six types of ablation variants in many core evaluation indicators, showing stronger generalization ability and performance stability. The results show that the multi‐dimensional feature fusion architecture based on ViT can effectively integrate multi‐dimensional heterogeneous information and has clear and quantifiable modeling gains.

#### 3.4.1 Performance Evaluation Results of the Full GAVR Model and Three Single‐Channel Ablation Variants

**FIGURE 4:**
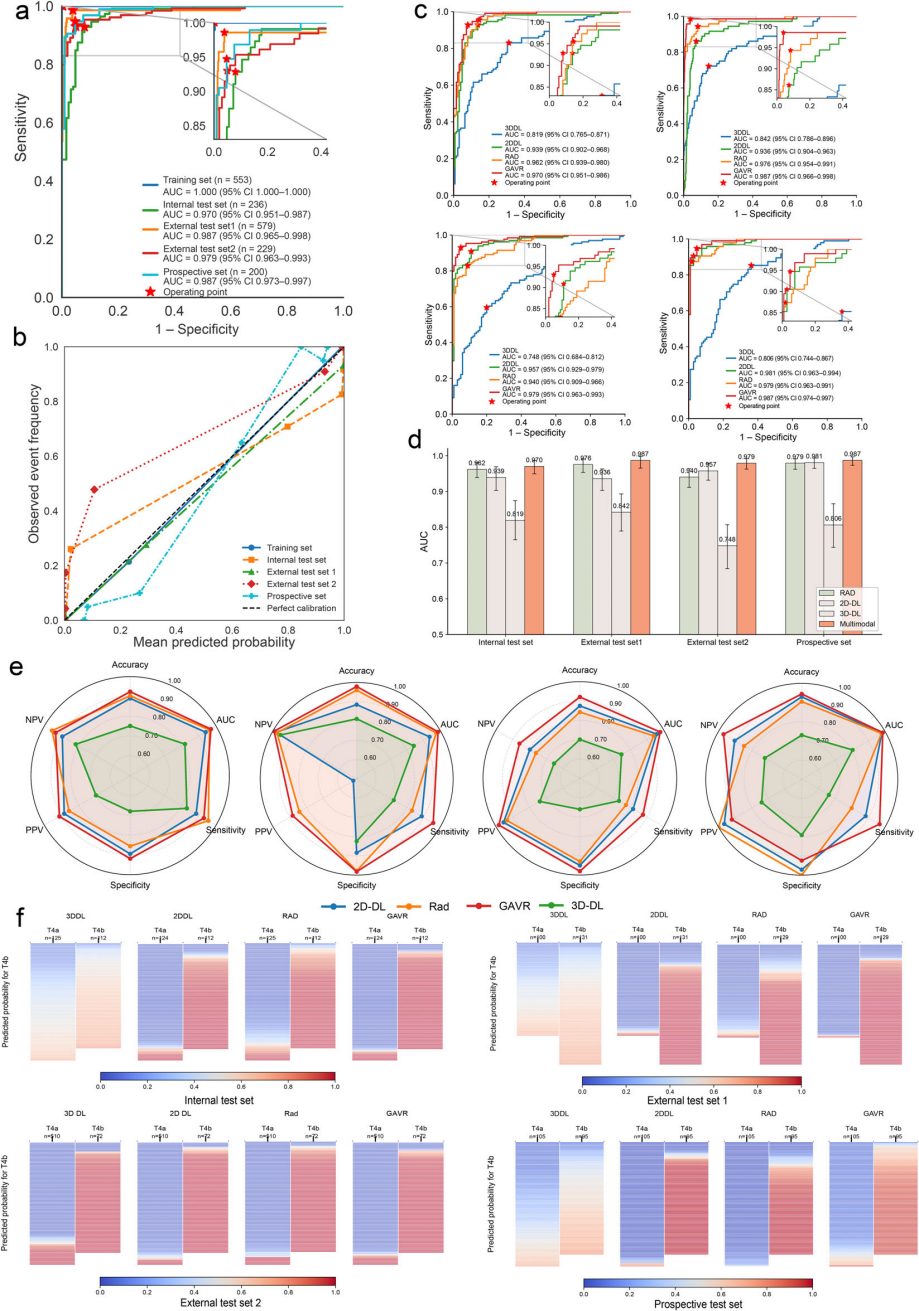
Comprehensive Performance Evaluation of the full GAVR model and the single‐channel ablation variant in a multicenter study cohort. (a) receiver operating characteristic (ROC) curves of the GAVR model in all five study cohorts; (b) global calibration curves of the GAVR model across all five study cohorts; (c) ROC curves of GAVR model versus three single‐channel ablation variants in four cohorts; (d) Bar plots of AUC of the three single‐channel ablation variants versus the full GAVR model in each of the four study cohorts (error bars indicate 95% confidence intervals); (e) Radar plot of six core evaluation indicators of each model in four validation cohorts (from left to right, Internaltestset, Externaltestset1, Externaltestset2 and Prospectiveset); (f) Heatmaps of case‐level prediction probability distribution of GAVR model and three types of single‐channel ablation variant models in four independent external validation cohorts. The horizontal axis is arranged by model groups, and the samples within each group are longitudinally blocked according to the true pathological T stage (T4a/T4b). Within each block, the samples are arranged in ascending order according to the predicted probability of T4b output by the model. A continuous warm chromatogram (red–blue) was used for color mapping, and the intensity of the color scale was positively correlated with the predicted probability: the warmer the color tone (the closer to Red), the higher the probability that the sample was judged as T4b by the model. The colder the color (closer to blue), the lower the probability.

There were three single‐channel ablation variants, namely radiomics only, 2D DL only, and 3D DL only. As shown in Table 3, all three single‐channel ablation variants showed basic discrimination ability in the gastric cancer T4a/T4b staging task, but their performance fluctuated significantly among different cohorts. In contrast, the full GAVR model consistently maintained optimal performance across all study cohorts (including the internal test set, the two external validation cohorts, and the prospective multicenter cohort) (Figure 4a), with low coefficients of variation of each index, which confirmed its robust generalization ability across centers. In contrast, the sensitivity, specificity, and AUC of the single‐channel model were generally decreased in the multicenter external validation cohort, especially in the Externaltestset2 cohort (Figure 4c,d), highlighting its sensitivity to shifting data distribution.

**TABLE 3:** Results of Performance comparison between the unimodal model and the GAVR model.

ModelName	Acc^a^	AUC^b^	95%CI^c^	Sensitivity	Specificity	PPV^d^	NPV^e^
**Trainingset (*n* = 553)**							
GAVR	0.998	1.000	0.9998–1.0000	1.000	0.996	0.997	1.000
Radiomics	0.969	0.997	0.9949–0.9990	0.965	0.974	0.975	0.963
2DDL	0.989	0.999	0.9966–1.0000	0.990	0.989	0.990	0.989
3DDL	0.825	0.887	0.8563–0.9147	0.903	0.740	0.790	0.875
**Internaltestset(n = 237)**							
GAVR	0.924	0.970	0.9496–0.9866	0.929	0.919	0.912	0.934
Radiomics	0.903	0.962	0.9392–0.9806	0.955	0.856	0.856	0.955
2DDL	0.890	0.939	0.9030–0.9691	0.884	0.895	0.884	0.895
3DDL	0.751	0.819	0.7650–0.8742	0.830	0.680	0.699	0.817
**Externaltestset1(*n* = 583)**							
GAVR	0.979	0.987	0.9652–0.9980	0.958	0.982	0.883	0.994
Radiomics	0.960	0.976	0.9535–0.9909	0.831	0.978	0.843	0.977
2DDL	0.885	0.936	0.9031–0.9631	0.889	0.884	0.520	0.983
3DDL	0.811	0.842	0.7893–0.8933	0.722	0.824	0.366	0.955
**Externaltestset2(*n* = 231)**							
GAVR	0.921	0.979	0.9626–0.9922	0.876	0.980	0.983	0.860
Radiomics	0.843	0.940	0.9116–0.9637	0.775	0.930	0.935	0.762
2DDL	0.875	0.958	0.9317–0.9790	0.817	0.950	0.955	0.798
3DDL	0.701	0.748	0.6843–0.8070	0.733	0.660	0.739	0.654
**Prospectiveset(*n* = 200)**							
GAVR	0.945	0.987	0.9733–0.9969	0.968	0.924	0.920	0.970
Radiomics	0.905	0.979	0.9621–0.9914	0.800	1.000	1.000	0.847
2DDL	0.930	0.981	0.9648–0.9937	0.884	0.971	0.966	0.903
3DDL	0.73	0.8063	0.7437–0.8658	0.6632	0.7905	0.7412	0.7217

^a^ Acc, Accuracy;

^b^ AUC, Area under curve;

^c^ CI, Confidence interval;

^d^ PPV, Positive predictive value;

^e^ NPV, Negative predictive value.

To further evaluate the clinical practicability of the model, we performed a comprehensive validation from three dimensions: discriminant performance, probability Calibration, and prediction stability. The performance of the model was systematically characterized by the Calibration Curve, Brier score, and visualization of the predicted probability distribution. The calibration curve of the GAVR model was close to the ideal diagonal, indicating that the output probability of the GAVR model was highly consistent with the true event rate (see Figure 4b). This conclusion was further supported by the Brier scores, which showed that the GAVR model had the lowest Brier values (0.037 to 0.061) in four of the cohorts, which was significantly better than all the single‐channel ablation models (Table S5 in the Supporting Appendix), confirming its better probability fitting accuracy. In addition, the radar chart (Figure 4e) showed that the GAVR model showed excellent performance in all the key evaluation indicators, and the overall discrimination ability was more robust. The heatmap of case‐level prediction probability distribution (Figure 4f) visually reflected the great advantages of each model in the clarity of the discrimination boundary, the degree of probability separation between categories, and the consistency of the prediction confidence distribution for T4a/T4b subtypes.

#### 3.4.2 Performance Evaluation Results of the Full GAVR Model and the Three Dual‐Channel Combination Ablation Variants

**FIGURE 5:**
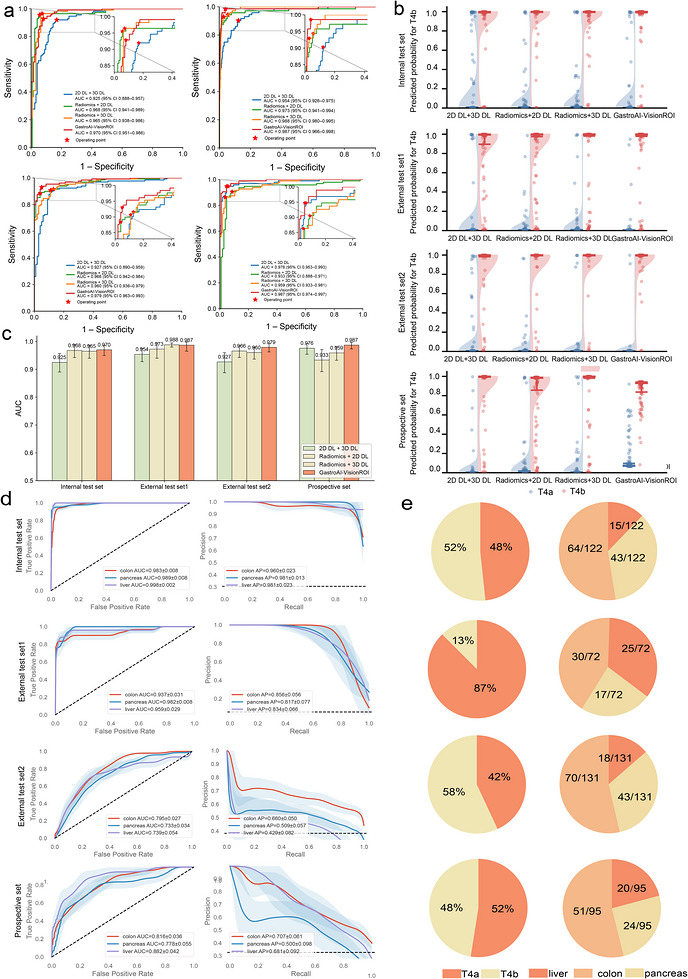
Performance of the GAVR model as compared with dual‐channel combination ablation Variants in a multicenter cohort and in the context of organ‐specific invasion. (a) Receiver operating characteristic (ROC) curves of the GAVR model and dual‐channel combination ablation variants in the training set, internal test set, external test set 1, external test set 2, and prospective test set; (b) Violin plot of predicted probability distribution of the GAVR model and dual‐channel combination ablation variants in different stages. The blue part on the left shows the predicted probability distribution of T4a cases, and the red part on the right shows the predicted probability distribution of T4b cases. (c) Bar graphs of AUC of the GAVR model and dual‐channel combination ablation variants in four external validation cohorts, which were used to visually compare the discrimination performance of different models in multicenter data. (d) ROC curves and precision‐recall (PR) curves for adjacent organ involvement (liver, pancreas, and colon) in four validation cohorts; (e) The proportion of patients with T4 gastric cancer subtypes (T4a and T4b) in the four validation cohorts (left column), and the proportion of patients with T4b involved organs (liver, pancreas, colon) (right column).

Table 4 summarizes the core evaluation indexes of the full GAVR model and the three dual‐channel combined ablation variants (Radiomics + 2DDL, Radiomics + 3DDL, 2DDL + 3DDL) on multiple datasets. As shown in Figure 5a, the receiver operating characteristic (ROC) curves of the GAVR model in each dataset were generally above those of the three types of dual‐channel ablation variants, indicating that its classification performance was more robust and consistent. The predicted probability distribution analysis shown in Figure 5b further revealed that the GAVR model had a clearer discrimination boundary for patients with T4a and T4b, and the overlap degree of the probability distribution between the two classes was significantly reduced, reflecting a stronger class discrimination ability. The AUC bar plot shown in Figure 5c visually confirms the continuous performance advantage of the GAVR model in multiple external validation cohorts. It is worth noting that although some dual‐channel ablation variants were close to the full GAVR model in terms of accuracy and AUC metrics in individual cohorts, for example, Radiomics + 2DDL and Radiomics + 3DDL in External Test Set 1 and 2DDL + 3DDL in Prospective Set. However, there are still significant differences in sensitivity between these two methods. In the rest of the validation cohorts, such variants showed significant performance fluctuations, especially a systematic decrease in sensitivity indicators. In summary, compared with all six types of ablation variants, the GAVR model effectively alleviated the tendency of overfitting and showed highly consistent discrimination efficiency and excellent cross‐center generalization ability in all validation cohorts.

**TABLE 4:** Results of Performance comparison between the bimodal model and the GAVR model.

ModelName	Acc^a^	AUC^b^	95%CI^c^	Sensitivity	Specificity	PPV^d^	NPV^e^
**Training set (*n* = 553)**							
GAVR	0.998	1.000	0.9998–1.0000	1.000	0.996	0.997	1.000
2DDL+3DDL	0.998	1.000	0.9999–1.0000	1.000	0.996	0.997	1.000
Rad^f^+2DDL	0.993	1.000	0.9996–1.0000	0.990	0.996	0.997	0.989
Rad+3DDL	0.993	1.000	0.9984–1.0000	0.993	0.993	0.993	0.993
**Internal test set(n = 237)**							
GAVR	0.924	0.970	0.9496–0.9866	0.929	0.919	0.912	0.934
2DDL+3DDL	0.869	0.925	0.8904–0.9564	0.920	0.823	0.824	0.919
Rad+2DDL	0.949	0.968	0.9423–0.9887	0.955	0.944	0.939	0.959
Rad+3DDL	0.945	0.965	0.9396–0.9861	0.955	0.936	0.930	0.959
**External test set1 (n = 583)**							
GAVR	0.979	0.987	0.9652–0.9980	0.958	0.982	0.883	0.994
2DDL+3DDL	0.897	0.954	0.9278–0.9751	0.833	0.906	0.556	0.975
Rad+2DDL	0.962	0.973	0.9399–0.9945	0.887	0.972	0.818	0.984
Rad+3DDL	0.967	0.988	0.9799–0.9947	0.930	0.973	0.825	0.990
**External test set2 (*n* = 231)**							
GAVR	0.921	0.979	0.9626–0.9922	0.876	0.980	0.983	0.860
2DDL+3DDL	0.870	0.927	0.8876–0.9595	0.840	0.910	0.924	0.813
Rad+2DDL	0.904	0.966	0.9424–0.9840	0.845	0.980	0.982	0.831
Rad+3DDL	0.869	0.960	0.9367–0.9795	0.798	0.960	0.963	0.787
**Prospective set(*n* = 200)**							
GAVR	0.945	0.987	0.9733–0.9969	0.968	0.924	0.920	0.970
2DDL+3DDL	0.925	0.976	0.9542‐00.9930	0.874	0.971	0.965	0.895
Rad+2DDL	0.885	0.933	0.8917–0.9712	0.811	0.952	0.939	0.848
Rad+3DDL	0.895	0.959	0.9326–0.9808	0.821	0.962	0.951	0.856

^a^ Acc, Accuracy;

^b^ AUC, Area under curve;

^c^ CI, Confidence interval;

^d^ PPV, Positive predictive value;

^e^ NPV, Negative predictive value;

^f^ Rad, Radiomics.

Systematic ablation experiments were conducted to evaluate the contribution of each component of the proposed model. Results indicated that all ablated variants exhibited varying degrees of performance degradation across multiple external validation cohorts, primarily characterized by a systematic decrease in classification accuracy or significant fluctuations in discriminative metrics (e.g., AUC) in at least one external cohort. In contrast, the full‐architecture GAVR model maintained consistently high and stable discriminative performance across all external validation cohorts (Accuracy = 0.948 ± 0.029, AUC = 0.984 ± 0.005), thereby demonstrating its superior generalization ability and potential for clinical deployment. These findings provide robust empirical evidence supporting the necessity and structural irreplaceability of the multimodal collaborative modeling strategy proposed in this study (see Tables S5 and S6 for detailed results).

#### 3.4.3 Diagnostic Performance Analysis of GAVR Model for Gastric Cancer Invasion of Different Adjacent Organs

As shown in Figure 5d,e, the AUC of the model in different organ invasions ranged from 0.77 to 0.99, maintaining a high discrimination ability overall. Among them, the model had the best performance in identifying invasion of the liver, followed by the colon and pancreas. Although there are some performance differences among different organs, the GAVR model shows a stable ability to identify all types of invasion patterns. Detailed data are provided in Table S8.

### 3.5 Reader's Study

**FIGURE 6:**
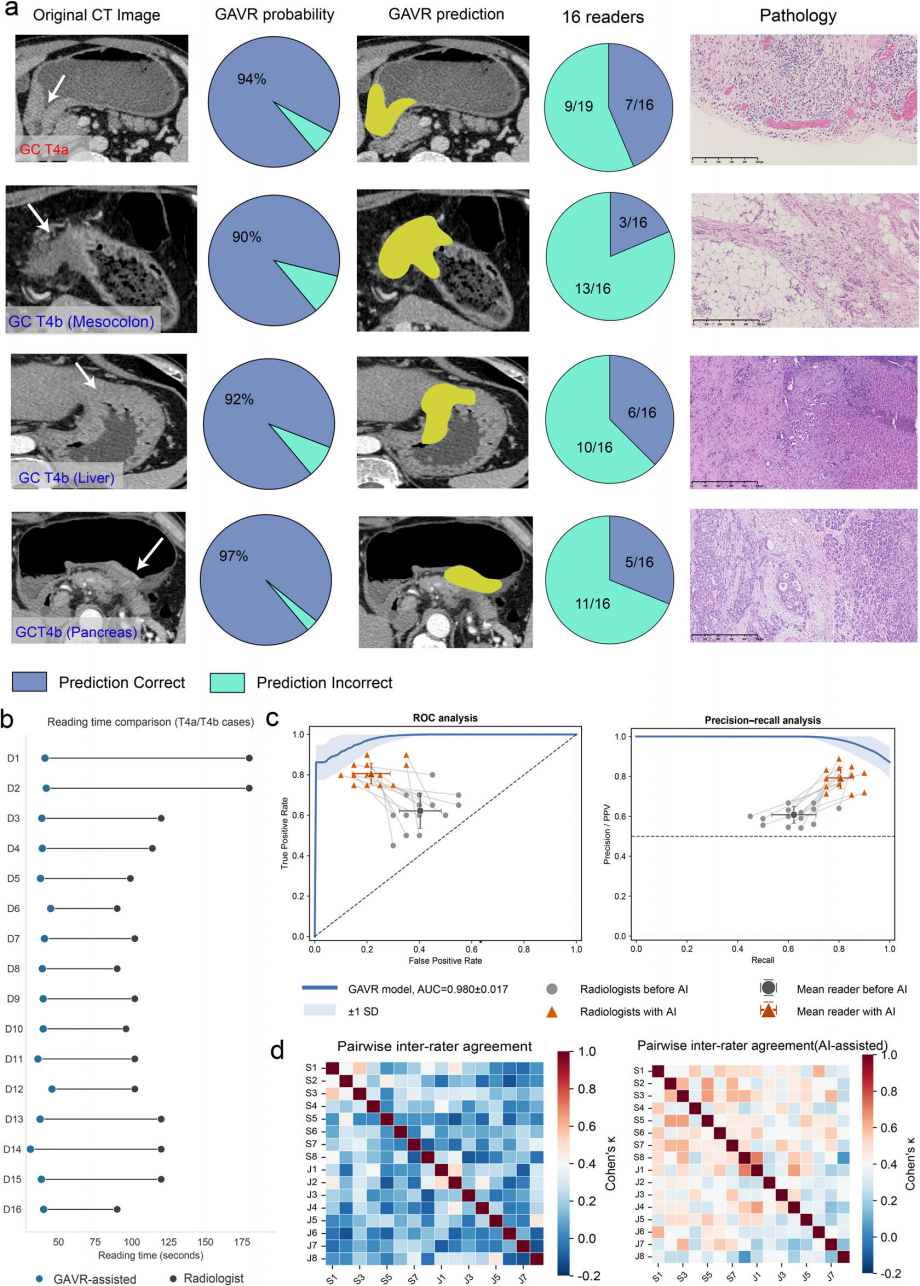
Comparative analysis and interpretability of the GAVR model for radiologists' interpretation performance. (a) Visualization of typical cases. The original CT images of patients with T4a and T4b (involving different adjacent organs), pie charts of probability distribution predicted by the GAVR model, prediction results of the model and areas of concern (the yellow mask indicates high‐response areas of the model), pie charts of probability distribution interpreted by 16 clinicians, and corresponding pathological results were presented in turn. (b) The reading time performance of 16 radiologists before and after the assistance of the model is shown. Each point represents the average reading time of a single radiologist on all samples; (c) ROC and precision‐recall (PR) analysis. The solid blue line shows the average ROC/PR curve of the GAVR model, and the light blue shading indicates the ± 1 standard deviation range obtained from 1000 bootstrap resamplings. The gray dots and orange triangles represent the operation points of 16 radiologists before and after AI assistance, respectively. The gray lines represent the changes in the performance of the same radiologist before and after AI assistance. The large dark markers and error bars indicate the average performance and standard deviation of the group of doctors; (d) Heat maps of Cohen's kappa statistic before and after GAVR model‐assisted radiologists to evaluate the inter‐clinician agreement.

In the image interpretation task simulating a standardized clinical workflow, the GAVR model showed superior ability to identify subtypes of cT4b gastric cancer than radiologists. The results of autonomous prediction were more consistent with the postoperative pathological diagnosis. The typical case analysis is shown in Figure 6a. This performance advantage was further validated by the interpretability analysis of Grad‐CAM (Figure S4 in the Appendix shows the visualized heat map of the key regions of the model's decision). Table 5 systematically compares the overall performance of three diagnostic modes: independent discrimination by the GAVR model, independent diagnosis by radiologists without assistance, and collaborative diagnosis by radiologists with the assistance of the GAVR model. The results showed that the accuracy of independent discrimination of the GAVR model was 0.925; The average diagnostic accuracy of 16 radiologists without assistance was 0.609 (95%CI: 0.586‐0.633), and the sensitivity and specificity were 0.622 and 0.597, respectively, suggesting that there was a high risk of missed diagnosis and misdiagnosis in cT4b lesions identification. With the assistance of the GAVR model, the physicians' interpretation time per CT image was significantly shortened from 100 to 175 s at baseline to less than 50 s on average, with a decrease of about 60% (Figure 6b), and the overall diagnostic accuracy was improved to 0.795 (95%CI: 0.774‐0.817), with a sensitivity of 0.806 and a specificity of 0.784 (Figure 6c). In addition, Cohen's kappa analysis showed that without the assistance of GAVR model, the mean value of the kappa coefficient of pairwise pairs of 16 radiologists was 0.219 (95%CI: 0.178–0.259), indicating a low level of agreement (Figure 6d, left panel). With the assistance of the GAVR model, the mean value was significantly increased to 0.591 (95%CI: 0.553–0.628), which reached a moderate consistency level (right panel of Figure 6d). The above results show that the GAVR model can not only significantly improve the recognition accuracy and interpretation efficiency of cT4b gastric cancer, but also effectively reduce the diagnostic dispersion caused by individual differences, so as to enhance the reliability, consistency, and robustness of clinical diagnosis.

**TABLE 5:** Diagnostic performance of radiologists with and without AI assistance for T4a/T4b classification.

Metric	Accuracy (95% CI)^a^	Specificity (95% CI)	Sensitivity (95% CI)	PPV (95% CI)^b^	NPV (95% CI)^c^
**GAVR**	0.925 (0.849–1.000)	0.900 (0.750–1.000)	0.950 (0.842–1.000)	0.905 (0.765–1.000)	0.947 (0.833–1.000)
**Radiologists Alone (RA)**	0.609 (0.586–0.637)	0.597 (0.554–0.640)	0.622 (0.575–0.669)	0.608 (0.585–0.631)	0.615 (0.587–0.643)
**Radiologists assisted by the GAVR (RABG)**	0.795 (0.774–0.817)	0.784 (0.746–0.823)	0.806 (0.779–0.834)	0.792 (0.764–0.821)	0.804 (0.781–0.827)
**∆ _RABG‐RA_** ^d^	0.186	0.187	0.184	0.184	0.189
***p* value**	< 0.001	< 0.001	< 0.001	< 0.001	< 0.001

^a^ CI, Confidence interval;

^b^ PPV, Positive predictive value;

^c^ NPV, Negative predictive value;

^d^ ΔRABG‐RA, Difference in performance metrics between radiologists assisted by the GAVR model and radiologists alone.

## 4 Discussion

Accurate preoperative cT staging is not only the prerequisite for formulating individualized treatment plans, but also the key clinical link to avoid the double risks of excessive intervention (such as unnecessary combined organ resection) and insufficient intervention (such as delaying systematic treatment of potentially transformable cases) [23].At present, high‐precision preoperative identification of T4a/T4b subtypes based on routine enhanced CT images is still one of the important bottlenecks affecting the quality of clinical decision‐making [24]. The main limitations of previous artificial intelligence models in T‐stage discrimination of gastric cancer are insufficient feature representation ability, single spatial modeling dimension, and poor cross‐center generalization performance. The AUC of the classification model constructed by Sun et al. based on two‐dimensional CT texture features was only 0.75, suggesting that the discriminative power of pure texture information on the boundary of deep invasion was limited [25]. The model established by Liu et al., using enhanced CT radiomics, had an AUC of 0.86 in distinguishing pT4b from non‐Pt4b, but did not integrate three‐dimensional spatial geometric features and key clinical covariates, and had obvious limitations in identifying complex T4 lesions (such as microinvasion and interface fibrosis) [16]. Although the model constructed by Tao et al. using 3D CNN increased the AUC to 0.81, it failed to achieve the refined differentiation of T4a and T4b subtypes [21]. Based on eROI analysis, the GAVR model proposed in this study innovatively encodes traditional radiomics features, two‐dimensional convolutional features, and three‐dimensional voxel‐based depth features into a multimodal token sequence. Relying on the unique self‐attention mechanism and cross‐modal attention mechanism of ViT architecture [26, 27], the dynamic interaction and adaptive weight allocation of multi‐source features at the global scale are realized. Thus, the spatial dependence and the heterogeneity of the interface microenvironment between the tumor body and its adjacent organs are effectively captured. In the multi‐center validation covering 200 prospective cohorts, GAVR was significantly better than the existing similar methods in the core indicators such as accuracy (ACC), AUC, and external generalization performance [28].

In order to further evaluate the clinical practicability of the GAVR model, this study conducted a prospective reader study to systematically investigate its effect on improving the interpretation efficiency of radiologists. The results showed that GAVR improved the accuracy of radiologists in diagnosing T4 subtypes by 18.5% and shortened the reading time by about 60%. The significant improvement in the sensitivity of GavR helps to reduce the unplanned combined organ resection caused by the misclassification of cT3/cT4a, thereby reducing the incidence of perioperative complications and the risk of postoperative dysfunction.

In this paper, a typical case of cT4b rediscrimination in a prospective cohort was selected to explain the clinical value of the GAVR model in optimizing preoperative cT staging and supporting individualized treatment decisions. A 62‐year‐old male patient was diagnosed with poorly differentiated adenocarcinoma in the greater curvature of the antrum of the stomach. The GAVR model was judged to be cT4b based on its enhanced CT images. The result of gastroscopy combined with endoscopic ultrasonography was cT3 (tumor infiltration into the subserosal layer). Conventional enhanced CT images showed that there was a fat gap between the tumor and the pancreatic head, so the initial imaging judgment supported direct surgical intervention. After discussion by the Multidisciplinary Team (MDT), we decided to perform diagnostic laparoscopic exploration first. During the operation, it was confirmed that the tumor was tightly adhered to the head of the pancreas and could not be safely separated. Therefore, the radical resection plan was aborted, and neoadjuvant chemotherapy was started. Imaging evaluation after treatment showed that the tumor was significantly regressed. Second laparoscopic exploration showed that the original adhesion area had transformed into a loose fibrous membrane structure, which could be removed by gentle and blunt separation. Frozen section of the pancreatic capsule and superficial tissue obtained from the original dense adhesion site showed hyperplastic fibrous connective tissue with hyalinization and a small amount of chronic inflammatory cell infiltration, but no malignant tumor cells. This pathological result highly suggested that neoadjuvant therapy had induced a deep pathological response, and the previously suspected cT4b invasion area had achieved a fibrotic replacement cancer‐free state.

The accurate identification of cT4b by the GAVR model effectively makes up for the inherent limitations of traditional imaging interpretation in the identification of micro‐invasion signs at the tumor‐adjacent organ interface. It not only avoids unnecessary combined organ resection but also reduces the risk of intraperitoneal free cancer cell dissemination and perioperative complications. In summary, the GAVR model not only significantly outperforms the existing radiomics methods and conventional image interpretation level in diagnostic performance [29, 30], but also relies on the enhanced Region‐of‐Interest (eROI) multi‐dimensional feature fusion and global spatial modeling strategy. It provides a refined preoperative staging decision support tool with clinical transformation potential for locally advanced gastric cancer.

This study still has limitations. First, the current model uses the cT4a/cT4b binary classification framework, which is still insufficient to support treatment decisions in clinical practice: In addition to integrating N and M stages, some high‐risk cT4b cases (such as mesenteric root invasion, celiac trunk entrapment or main portal vein trunk) are difficult to achieve R0 resection even after systemic treatment, suggesting that a multi‐dimensional classification model including resectability prediction should be constructed in the future. Second, the interpretability of the decision‐making process of the model needs to be deepened: Although this study combined t‐SNE dimensionality reduction visualization and SHAP value analysis to quantitatively evaluate the contribution of multimodal features, the above methods still belong to the category of post‐hoc interpretation and cannot fully reveal the hierarchical feature abstraction and reasoning path within ViT. Third, the External validation cohort was all from the tertiary hospitals in northern China, which may have regional and population‐specific biases, which may be the reason for the lowest ACC of GAVR model in External test set 2. Follow‐up studies need to include data covering multiple regions, multi‐ethnic groups and international cohorts in order to comprehensively evaluate and improve the universality and robustness of the model.

## 5 Conclusions

In this study, a GAVR model based on multi‐dimensional feature deep learning was proposed and validated to address the key clinical challenges in CT image analysis of gastric cancer. The model adopts the eROI multi‐dimensional feature extraction strategy and adopts the ViT fusion architecture to effectively deal with problems such as the heterogeneity of the peritumoral microenvironment and the strong subjectivity of manual interpretation of T4 stage tumor microinvasion. Based on the validation of a multicenter cohort of 1804 patients with a total of 13 048 CT images, the GAVR model showed excellent diagnostic performance and robust cross‐institution generalization ability. At the clinical level, the GAVR model can significantly improve the accuracy and work efficiency of radiologists in the preoperative identification of T4a/T4b substages. As a noninvasive and objective decision‐making tool, GAVR is expected to be integrated into the multidisciplinary diagnosis and treatment process of gastric cancer, support the formulation of individualized surgical strategies, reduce the rate of unnecessary combined organ resection, and improve the prognosis of patients.

## Author Contributions

G.Z., X.C., and P.J. contributed equally to this work. J.Z., G.Z., and X.C. conceived and designed the study. G.Z., X.C., and P.J. developed the methodology and led the software development and algorithm implementation. X.X., Y.L. (**Yingjie Li**), J.W., Y.H., and N.Z. collected and curated the clinical and imaging data. X.X., Y.L. (**Yan Li**), J.W., C.Y., and Z.Y. conducted the investigation, including data collection, image annotation, and case verification. P.J., Y.L. (Yingjie Li), X.Z., B.Q., H.W., H.Z., and Y.L. (Yan Li) performed model validation, performance testing, and external dataset harmonization. X.C., C.Y., J.Z. (**Jingyu Zhang**), Q.Z., Y.H., N.Z., F.L., and H.Z. conducted the formal statistical analysis. X.Z., Y.Z., W.M., and J.Z. (**Jing Zhang**) supervised the study. G.Z. and J.Z. (Jing Zhang) acquired the funding. J.W., J.Z. (Jingyu Zhang), Q.Z., F.L., B.Q., H.W., and Y.L. (Yan Li) prepared the visualizations and figures. Y.Z. and X.Z. were responsible for project administration and coordination. Y.Z., W.M., and X.Z. Provided resources and institutional support. G.Z., X.C., and P.J. drafted the original manuscript. J.Z. (Jing Zhang), W.M., Y.Z., and X.Z. Reviewed and edited the manuscript and critically revised its intellectual content. All authors had full access to all study data, took responsibility for the integrity of the data and the accuracy of the data analysis, and approved the final version of the manuscript.

## Funding

This work was supported by the Liaoning Provincial Natural Science Foundation (Grant Nos. 2024‐MS‐269 and 2024‐MS‐264), the Tianjin Key Medical Discipline Construction Project (Grant No. TJYXZDXK‐3‐003A), the Tianjin Science and Technology Major Project and Engineering, Public Health Youth Project (25ZXWQSY00060), and the Liaoning Province Science and Technology Joint Plan (Grant No. 2023JH2/101700195).

## Conflicts of Interest

The authors declare no conflicts of interest.

## Supporting information




**Supporting File**: advs76455‐sup‐0001‐SuppMat.docx.

## Data Availability

The data that support the findings of this study are openly available in GAVR‐Gastric‐T4‐Staging at https://github.com/18846068128/GAVR‐Gastric‐T4‐Staging, reference number 10.5281/zenodo.18364938.
