# Supplementary material for: Multiscale Spatial Fusion Feature‐Driven Characterization of Gastric Cancer Invasive Margins: A Multicenter Cohort Study for Preoperative Accurate Differentiation Between T4a and T4b Subtypes
Source: Adv Sci (Weinh). 2026 Jul 20:e76455. Online ahead of print. doi: 10.1002/advs.76455 (PMC13384034; doi:10.1002/advs.76455)
Supplement: Supplementary file 1 — Supporting File: advs76455‐sup‐0001‐SuppMat.docx. [file ADVS-9999-e76455-s001.docx]

**Supplementary information**

**Multiscale Spatial Fusion Feature-Driven Characterization of Gastric Cancer Invasive Margins: A Multicenter Cohort Study for Preoperative Accurate Differentiation Between T4a and T4b Subtypes**

*Guoliang Zheng¹‡, Xiaomiao Chai²‡, Peng Jin³‡, Xin Xin^4^, Yingjie Li^5^, Jia Wei², Chun Yang², Zishuo Yan², Jingyu Zhang^6^, Qianning Zhao^7^,* *Yingchen Han^6^, Ning Zhang^8^, Fuze Li^1^, Bo Qiao¹, Huan Wang^9^, Huachuan Zheng^10^, Yan Li^11^, Xin Zhang^12^*, Yan Zhao^1^*, Wenjun Mao^13^*, Jing Zhang²**

**Affiliations**

^1^ Department of Gastric Surgery, Cancer Hospital of Dalian University of Technology, Liaoning Cancer Hospital & Institute, Shenyang 110042, Liaoning, China

^2^ School of Pharmacy, China Medical University, Shenyang 110122, Liaoning, China

^3^ Department of Gastric Surgery, Tianjin Medical University Cancer Institute and Hospital, National Clinical Research Center for Cancer, Tianjin Key Laboratory of Digestive Cancer, Tianjin Clinical Research Center for Cancer, Tianjin 300060, China

^4^ Shenyang Mental Health Center, Shenyang 110168, Liaoning, China

^5^ Department of Radiology, Hunnan Central Hospital, Shenyang 110014, Liaoning, China

^6^ Department of Medical Oncology, The First Hospital of China Medical University, Shenyang 110001, Liaoning, China

^7^ School of Management, Hebei University, Baoding 071000, Hebei, China

^8^ Department of Pathology, Cancer Hospital of Dalian University of Technology, Liaoning Cancer Hospital & Institute, Shenyang 110042, Liaoning, China

^9^ Department of Radiotherapy, Cancer Hospital of China Medical University, Liaoning Cancer Hospital & Institute, Cancer Hospital of Dalian University of Technology, Shenyang 110042, Liaoning, China.

^10^ Center of Translational Medicine and Department of Gastroenterology, The First Affiliated Hospital of Jinzhou Medical University, Jinzhou 121001, Liaoning, China

^11^ Department of Laboratory Medicine, Institute of Laboratory Medicine, Sichuan Provincial People’s Hospital, School of Medicine, University of Electronic Science and Technology of China, Chengdu 610072, Sichuan, China

^12^ Department of Nuclear Medicine, Shengjing Hospital of China Medical University, Shenyang 110004, Liaoning, China

^13^ Department of Thoracic Surgery, The Affiliated Wuxi People’s Hospital of Nanjing Medical University, Wuxi Medical Center, Nanjing Medical University, Wuxi 214023, Jiangsu China

**Correspondence to**

**Xin Zhang** (E-mail:20092315@cmu.edu.cn)
**Yan Zhao** (E-mail: drzhao@dlut.edu.cn)
**Wenjun Mao (**E-mail: maowenjun1@njmu.edu.cn)
**Jing Zhang** (E-mail: [zhangjing@cmu.edu.cn](mailto:zhangjing@cmu.edu.cn))

**Section S1**. CT image protocols and images preprocessing

**Section S2**. Model Parameter Configuration

**Section S3.** Internal Architecture and Hyperparameters of the GastroAI-VisionROI Model

**Table S1.** CT acquisition parameters across three centers. Minor variations exist in scanner models, contrast injection protocols, and image reconstruction settings

**Table S2.** Performance comparison of five CNN architectures in the training and internal test sets

**Table S3.** Performance comparison of five 3D deep learning architectures in the training and internal test sets

**Table S4.** Name initials and qualifications of the readers

**Table S5**. Cross-cohort accuracy statistics of the complete GAVR model and structured ablation variants across external and prospective validation cohorts

**Table S6**. Pairwise accuracy comparisons between the complete GAVR model and structured ablation variants across external and prospective validation cohorts

**Table S7.** Calibration performance of five models across five datasets (Brier score comparison)

**Table S8.** Performance Analysis of GAVR in Predicting Gastric Cancer Invasion of Different Organs

**Figure S1.** Ablation Study Evaluating the Effectiveness of the Transformer-based Fusion Framework

**Figure S2.** Impact of ROI Expansion and Multidimensional Feature Analysis on Model Performance

**Figure S3.** Segmentation Results Visualization

**Figure S4.** Grad-CAM Visualization of the Decision-Making Process of the GAVR Model

**Section S1. CT image protocols and images preprocessing**

All enrolled patients from the three participating centers—Liaoning Cancer Hospital, Shengjing Hospital of China Medical University, and Zhejiang Cancer Hospital—underwent contrast-enhanced CT (CECT) examinations. Although acquisition protocols were broadly comparable among centers, variations in scanner models and specific imaging parameters were present (summarized in Table S1).

Before scanning, patients fasted for at least 4–6 hours and ingested 600–1000 mL of warm water to achieve adequate gastric distension. CT scans were performed with full coverage of the stomach region during a single breath-hold.

For CECT, a nonionic iodinated contrast agent with a concentration of 320–350 mg I/mL was injected intravenously at a rate of 2.5–3.0 mL/s. The contrast dose varied across centers (60 mL at Liaoning Cancer Hospital and 80 mL at the other two centers). Portal venous phase images were acquired with scan delays ranging from 45–70 seconds, depending on the institution.

Scanner models included Toshiba and Philips (including Philips Ingenuity) systems, operated at a tube voltage of 120 kV with automatic tube current modulation. Detector collimation ranged from 0.5 × 64 to 0.625 × 128, with an image matrix of 512 × 512. The reconstructed slice thickness varied slightly among centers, ranging from 1 mm to 5 mm.

To minimize inter-institutional variability caused by differences in imaging equipment and acquisition protocols, all CT images underwent standardized preprocessing. Specifically:

(1)CT images were resampled to an isotropic voxel size of 1×1×1 mm³ using cubic spline interpolation;

(2)Pixel intensity values were normalized to a range of –1024 to 1024 HU, with abdominal CT windowing parameters (WL = 50, WW = 350);

(3)Data augmentation strategies were applied during model training, including random rotations (0°–360°), flipping, and variations in brightness, contrast, and saturation.

During the inference phase, a unified preprocessing pipeline was used to ensure consistency in model predictions regardless of scanner or acquisition differences.

**Section S2. Model Parameter Configuration**

A two-dimensional U-Net (2D U-Net) architecture was developed for automatic segmentation of gastric cancer lesions. The model was implemented in PyTorch (v2.0) and trained on an NVIDIA GPU platform.

(1) Network Architecture

The U-Net model adopted a standard encoder–decoder structure, taking a single-channel 2D CT slice (1×256×256) as input and producing a binary segmentation mask (1×256×256) as output.

The detailed network configuration is summarized below:

| **Module** | **Layer composition** | **Channels** | **Kernel size** | **Activation** |
| --- | --- | --- | --- | --- |
| Encoder 1 | Conv2d ×2 | 1 → 32 | 3×3 | ReLU |
| Encoder 2 | Conv2d ×2 | 32 → 64 | 3×3 | ReLU |
| Encoder 3 | Conv2d ×2 | 64 → 128 | 3×3 | ReLU |
| Bottleneck | Conv2d ×2 | 128 → 256 | 3×3 | ReLU |
| Decoder 3 | ConvTranspose2d + Conv2d ×2 | 256 → 128 | 2×2 / 3×3 | ReLU |
| Decoder 2 | ConvTranspose2d + Conv2d ×2 | 128 → 64 | 2×2 / 3×3 | ReLU |
| Decoder 1 | ConvTranspose2d + Conv2d ×2 | 64 → 32 | 2×2 / 3×3 | ReLU |
| Output layer | Conv2d + Sigmoid | 32 → 1 | 1×1 | Sigmoid |

The final output was activated by a Sigmoid function to generate a probabilistic segmentation map, which was thresholded to obtain binary tumor masks.

(2) Data Preprocessing and Normalization

All CT volumes were loaded from .nii.gz files using Nibabel and sliced along the axial plane. Each 2D slice was normalized to the range [0,1] and resized to 256×256 pixels to ensure uniform input dimensions.
The preprocessing steps included:

- Intensity normalization:

$$I_{norm}=\frac{I-I_{min}}{I_{max}-I_{min}}$$

- Mask binarization: Voxels with values >0 were considered tumor regions.
- Spatial resampling: Performed using transforms.Resize((256,256)) from the PIL library.

(3) Training Configuration

| **Parameter** | **Value / Setting** |
| --- | --- |
| Optimizer | Adam |
| Learning rate | 1×10⁻⁴ |
| Loss function | Binary Cross-Entropy (BCE) |
| Batch size | 2 |
| Epochs | 100 (1 epoch shown in the code example for demonstration) |
| Input channels | 1 (grayscale CT) |
| Output channels | 1 (binary mask) |
| Weight initialization | Xavier normal |
| Activation functions | ReLU (hidden layers), Sigmoid (output) |
| Pooling layer | MaxPooling (stride=2) |
| Upsampling layer | ConvTranspose2d (stride=2) |

(4) Model Saving and Inference

After training, model weights were saved in .pth format at the following path:

tumor_seg_model.pth

The trained segmentation model was subsequently applied to independent test cohorts for automatic tumor segmentation on unannotated CT scans, enabling standardized ROI cropping and consistent input regions for downstream feature extraction and model training.

**Section S3. Internal Architecture and Hyperparameters of the GastroAI-VisionROI Model**

The GastroAI Vision-based ROI (GAVR) model is a multimodal deep learning framework designed to integrate heterogeneous information sources, including radiomics features, 2D deep learning representations, and 3D deep learning representations, for the classification of gastric cancer T4a versus T4b stages. The internal architecture consists of three major components: modality-specific embedding, transformer-based multimodal fusion, and classification.

(1) Modality-specific Feature Embedding

Each modality is first processed by an independent embedding branch that projects the original feature vectors into a shared latent space.

For a modality *m*, the embedding process is defined as:

$$E_{m}=\text{Dropout}(\text{ReLU}(W_{m}X_{m}+b_{m}))$$

where:

- $X_{m}$denotes the input feature vector for modality *m*
- $W_{m}$and $b_{m}$are learnable parameters
- ReLU is used as the nonlinear activation function
- Dropout with probability 0.3 is applied to reduce overfitting

Embedding dimensions for each modality are defined as follows:

| **Modality** | **Embedding Dimension** |
| --- | --- |
| Radiomics features | 64 |
| 2D deep learning features | 64 |
| 3D deep learning features | 64 |

These embedded representations are treated as modality tokens for the subsequent transformer-based fusion module.

(2) Token Construction and Positional Encoding

The modality embeddings are arranged as a token sequence:

$$T=\{T_{rad},T_{2D},T_{3D}\}$$

where each token corresponds to one modality.

To preserve token identity and allow the transformer to distinguish different modalities, a learnable positional embedding $P$is added:

$$Z_{0}=T+P$$

where $Z_{0}$represents the input token sequence to the transformer encoder.

(3) Transformer-based Multimodal Fusion

Cross-modal interactions are modeled using a Transformer encoder composed of multiple attention blocks.

Each transformer block consists of two main components:

Multi-Head Self-Attention (MHSA)

The attention mechanism is defined as:

$$\text{Attention}(Q,K,V)=\text{softmax}\left( \frac{QK^{T}}{\sqrt{d}} \right)V$$

where $Q$, $K$, and $V$denote the query, key, and value matrices derived from the token representations.

Multiple attention heads are used to capture different types of cross-modal relationships.

Feed-Forward Network (FFN)

The attention outputs are passed through a position-wise feed-forward network:

$$\text{FFN}(x)=\text{ReLU}(W_{1}x+b_{1})W_{2}+b_{2}$$

Residual connections and layer normalization are applied to stabilize training:

$$Z^{'}=\text{LayerNorm}(Z+\text{MHSA}(Z))$$

$$Z_{out}=\text{LayerNorm}(Z^{'}+\text{FFN}(Z^{'}))$$

This design enables the model to learn adaptive weighting across modalities and capture global dependencies among radiomics and imaging features.

(4) Classification Head

After transformer fusion, the output token representations are aggregated using global average pooling and passed to a fully connected classifier:

$$y=\text{Softmax}(W_{c}Z+b_{c})$$

The classification head consists of:

- Fully connected layer (128 units)
- ReLU activation
- Dropout (0.5)
- Final linear layer producing probabilities for T4a and T4b classes

(5) Training Configuration

The model is optimized using cross-entropy loss:

$$L=-\sum y\log(\hat{y})$$

Training settings:

- Optimizer: Adam
- Initial learning rate: 0.001
- Weight decay: $1\times{10}^{-4}$
- Learning rate scheduler: StepLR (decay factor 0.1 every 10 epochs)
- Batch size: 32

The dataset is split into training and validation sets with a ratio of 7:3 using stratified sampling.

The model with the highest validation accuracy is selected as the final model for testing.

**Supplemental Tables**

**Table S1. CT acquisition parameters across three centers. Minor variations exist in scanner models, contrast injection protocols, and image reconstruction settings**

| **Parameter** | **Hospital 1 (Liaoning Cancer Hospital)** | **Hospital 2 (Shengjing Hospital of China Medical University)** | **Hospital 3 (Tianjin Medical University Cancer Institute and Hospital)** |
| --- | --- | --- | --- |
| Ct^a)^ Scanner | Toshiba | Philips | Philips Ingenuity |
| Tube Voltage [kV]^b)^ | 120 | 120 | 120 |
| Tube Current [mAs]^c)^ | Automatic | Automatic | Automatic |
| Detector Collimation | 0.5 × 64 | 0.625 × 64 or 0.625 × 128 | 0.625 × 128 |
| Contrast Agent^d)^ Concentration [mg I mL⁻¹]^e)^ | 320 | 350 | 350 |
| Contrast Agent Dose [ml]^f)^ | 60 | 80 | 80 |
| Injection Rate [mL s⁻¹]^g)^ | 3.0 | 2.5 | 3.0 |
| Venous Phase Scan Delay [s]^h)^ | 45–60 | 70 | 65–70 |
| Image Matrix^i)^ | 512 × 512 | 512 × 512 | 512 × 512 |
| Reconstructed Slice Thickness [mm]^j)^ | 1-5 | 3 | 1-2 |

a) CT, computed tomography; b) kV, kilovoltage; c) mAs, milliampere-seconds; d) Detector collimation, number and thickness of detector rows used for image acquisition (e.g., 0.5 × 64 indicates 64 detector rows with a thickness of 0.5 mm each); e) mg I mL⁻¹, iodine concentration of contrast agent; f) mL, milliliters; g) mL s⁻¹, contrast injection rate; h) s, seconds; i) Image matrix, pixel dimensions of reconstructed images (e.g., 512 × 512); j) mm, millimeters.

**Table S2. Performance comparison of five CNN architectures in the training and internal test sets**

| **Model** | **Acc^a)^** | **AUC^b)^** | **95% Ci^c)^** | **Sensitivity** | **Specificity** | **PPV^d)^** | **NPV^e)^** |
| --- | --- | --- | --- | --- | --- | --- | --- |
| **Training set (n = 553)** |  |  |  |  |  |  |  |
| ResNet50 | 0.77 | 0.86 | 0.8321–0.8938 | 0.71 | 0.84 | 0.83 | 0.73 |
| DenseNet121 (selected) | 0.8 | 0.89 | 0.8660–0.9179 | 0.74 | 0.87 | 0.86 | 0.76 |
| MobileNetV2 | 0.75 | 0.84 | 0.8122–0.8735 | 0.69 | 0.82 | 0.81 | 0.71 |
| InceptionV3 | 0.78 | 0.87 | 0.8436–0.9005 | 0.72 | 0.85 | 0.84 | 0.74 |
| VGG16 | 0.74 | 0.83 | 0.7987–0.8620 | 0.68 | 0.81 | 0.8 | 0.7 |
| **Internal test set (n = 237)** |  |  |  |  |  |  |  |
| ResNet50 | 0.81 | 0.87 | 0.8254–0.9171 | 0.82 | 0.8 | 0.8 | 0.82 |
| DenseNet121 (selected) | 0.84 | 0.89 | 0.8434–0.9281 | 0.85 | 0.82 | 0.81 | 0.86 |
| MobileNetV2 | 0.79 | 0.85 | 0.8043–0.8939 | 0.8 | 0.78 | 0.78 | 0.8 |
| InceptionV3 | 0.82 | 0.88 | 0.8349–0.9188 | 0.83 | 0.81 | 0.81 | 0.83 |
| VGG16 | 0.78 | 0.84 | 0.7926–0.8783 | 0.79 | 0.77 | 0.77 | 0.79 |

a) Acc, accuracy; b) AUC, area under the receiver operating characteristic curve; c) CI, confidence interval; d) PPV, positive predictive value; e) NPV, negative predictive value.

**Table S3**. **Performance comparison of five 3D deep learning architectures in the training and internal test sets**

| **Model** | **Acc^a)^** | **AUC^b)^** | **95% Ci^c)^** | **Sensitivity** | **Specificity** | **PPV^d)^** | **NPV^e)^** |
| --- | --- | --- | --- | --- | --- | --- | --- |
| **Training set (n = 553)** |  |  |  |  |  |  |  |
| ResNet3D | 0.83 | 0.9 | 0.8751–0.9247 | 0.84 | 0.82 | 0.83 | 0.83 |
| DenseNet3D | 0.85 | 0.91 | 0.8870–0.9329 | 0.86 | 0.84 | 0.85 | 0.85 |
| ShuffleNet3D (selected) | 0.87 | 0.93 | 0.9063–0.9495 | 0.88 | 0.86 | 0.87 | 0.87 |
| Transformer3D | 0.82 | 0.89 | 0.8614–0.9162 | 0.81 | 0.83 | 0.82 | 0.82 |
| OnekeyAI | 0.8 | 0.87 | 0.8423–0.9017 | 0.79 | 0.81 | 0.8 | 0.8 |
| **Internal test set (n = 237)** |  |  |  |  |  |  |  |
| ResNet3D | 0.76 | 0.85 | 0.8021–0.8924 | 0.83 | 0.7 | 0.72 | 0.82 |
| DenseNet3D | 0.78 | 0.87 | 0.8228–0.9066 | 0.85 | 0.72 | 0.73 | 0.84 |
| ShuffleNet3D (selected) | 0.79 | 0.88 | 0.8397–0.9232 | 0.87 | 0.73 | 0.74 | 0.86 |
| Transformer3D | 0.75 | 0.84 | 0.7963–0.8784 | 0.82 | 0.69 | 0.71 | 0.81 |
| OnekeyAI | 0.74 | 0.83 | 0.7845–0.8701 | 0.8 | 0.68 | 0.7 | 0.8 |

a) Acc, accuracy; b)AUC, area under the receiver operating characteristic curve; c) CI, confidence interval; d) PPV, positive predictive value; e) NPV, negative predictive value.

**Table S4**. **Name initials and qualifications of the readers**

| **Hospital** | **Location** | **Name (Initials)** | **Qualification** | **Professional Level** |
| --- | --- | --- | --- | --- |
| Xinhua Hospital Affiliated to Dalian University | Liaoning Province | Junping Gong | Attending Physician | Junior |
| Donggang Traditional Chinese Medicine Hospital | Liaoning Province | Xianchen Gao | Associate Chief Physician | Senior |
| Liaoning Provincial Third Rongjun Youfu Hospital | Liaoning Province | Yingchun Niu | Chief Physician | Senior |
| Fukuang General Hospital, Liaoning Health Industry Group | Liaoning Province | Hongbo Jia | Chief Physician | Senior |
| Liaohua Hospital, Liaoyang City | Liaoning Province | Lin Shen | Associate Chief Physician | Senior |
| Dawa District People’s Hospital, Panjin City | Liaoning Province | Xueli Guan | Associate Chief Physician | Senior |
| Panjin Central Hospital | Liaoning Province | Hua Zhao | Associate Chief Physician | Senior |
| The Fifth People’s Hospital of Shenyang | Liaoning Province | Gang Shen | Chief Physician | Senior |
| Hunnan District Central Hospital, Shenyang | Liaoning Province | Yingjie Li | Associate Chief Physician | Senior |
| Wafangdian Maternity and Child Health Hospital | Liaoning Province | Peng Wang | Associate Chief Physician | Senior |
| Wafangdian Maternity and Child Health Hospital | Liaoning Province | Kejia Wang | Associate Chief Physician | Senior |
| Wafangdian Second Hospital | Liaoning Province | Zhaoming Dai | Resident Physician | Junior |
| Wafangdian Kanghe Hospital | Liaoning Province | Zuofeng Fu | Associate Chief Physician | Senior |
| Yingkou Central Hospital | Liaoning Province | Rui Liu | Associate Chief Physician | Senior |
| Wafangdian Third Hospital Co., Ltd. | Liaoning Province | Chuang Liu | Chief Physician | Senior |
| Yingkou Central Hospital | Liaoning Province | Rui Liu | Associate Chief Physician | Senior |

**Table S5. Overall Performance Analysis of the Full GAVR Model and Its Structured Ablation Variants Across External and Prospective Validation Cohorts**

| **Model Variant** | **Mean Accuracy** | **Standard Deviation** | **Mean AUC** | **Standard Deviation** |
| --- | --- | --- | --- | --- |
| GAVR | 0.948 | 0.029 | 0.984 | 0.005 |
| Radiomics | 0.903 | 0.059 | 0.965 | 0.022 |
| 2DDL^a)^ | 0.897 | 0.029 | 0.958 | 0.023 |
| 3DDL^b)^ | 0.747 | 0.057 | 0.799 | 0.047 |
| 2DDL+3DDL | 0.897 | 0.028 | 0.952 | 0.025 |
| Radiomics +2DDL | 0.917 | 0.040 | 0.957 | 0.021 |
| Radiomics +3DDL | 0.910 | 0.051 | 0.969 | 0.016 |

a) 2DDL, two-dimensional deep learning model; b) 3DDL, three-dimensional deep learning model.

**Table S6. Pairwise accuracy comparisons between the complete GAVR model and structured ablation variants across external and prospective validation cohorts**

| **Pairwise Model Comparison** | **External test set1**  **ΔACC** | **P** | **External test set2**  **ΔACC** | **P** | **Prospective set ΔACC** | **P** |
| --- | --- | --- | --- | --- | --- | --- |
| GAVR vs Radiomics | 0.019 | 0.066 | 0.078 | 0.003 | 0.04 | 0.17 |
| GAVR vs 2D Dl^a)^ | 0.094 | <0.001 | 0.046 | 0.046 | 0.015 | 0.663 |
| GAVR vs 3D DL^b)^ | 0.168 | <0.001 | 0.22 | <0.001 | 0.215 | <0.001 |
| GAVR vs Radiomics + 2D DL | 0.017 | 0.055 | 0.017 | 0.453 | 0.06 | 0.002 |
| GAVR vs Radiomics + 3D DL | 0.012 | 0.096 | 0.052 | 0.019 | 0.05 | 0.066 |
| GAVR vs 2D DL + 3D DL | 0.082 | <0.001 | 0.051 | 0.031 | 0.02 | 0.522 |
| Radiomics + 2D DL vs Radiomics | 0.002 | 0.838 | 0.061 | 0.022 | −0.020 | 0.052 |
| Radiomics + 3D DL vs Radiomics | 0.007 | 0.584 | 0.026 | 0.327 | −0.010 | 0.823 |
| 2D DL + 3D DL vs 2D DL | 0.012 | 0.418 | −0.005 | 1 | −0.005 | 1 |
| Radiomics + 2D DL vs 2D DL | 0.077 | <0.001 | 0.029 | 0.19 | −0.045 | 0.004 |
| 3D DL + Radiomics vs 3D DL | 0.156 | <0.001 | 0.168 | <0.001 | 0.165 | <0.001 |
| 3D DL + 2D DL vs 3D DL | 0.086 | <0.001 | 0.169 | <0.001 | 0.195 | <0.001 |

a) 2DDL, 2D deep learning-only model; b) 3DDL, 3D deep learning-only model; c) ΔACC, difference in accuracy between paired models.

**Table S7. Calibration performance of five models across five datasets (Brier score comparison)**

| **Dataset** | **GastroAI-VisionROI** | **Radiomics** | **2DDL^a)^** | **3DDL^b)^** | **Clinical** |
| --- | --- | --- | --- | --- | --- |
| Training set（n=553) | 0.037 | 0.0879 | 0.136 | 0.1044 | 0.2288 |
| Internal test set(n=237) | 0.061 | 0.0989 | 0.1348 | 0.1468 | 0.2412 |
| External test set1(n=583) | 0.0584 | 0.1381 | 0.1005 | 0.1373 | 0.2462 |
| External test set2(n=231) | 0.117 | 0.086 | 0.2258 | 0.3271 | 0.2572 |
| Prospective set(n=200) | 0.2259 | 0.192 | 0.3895 | 0.255 | 0.2706 |

a) 2DDL, two-dimensional deep learning model; b) 3DDL, three-dimensional deep learning model.

**Table S8. Performance Analysis of GAVR in Predicting Gastric Cancer Invasion of Different Organs**

| **Model** | **Acc^a)^** | **AUC^b)^** | **95% Ci^c)^** | **Sensitivity** | **Specificity** | **PPV^d)^** | **NPV^e)^** |
| --- | --- | --- | --- | --- | --- | --- | --- |
| **Training set** |  |  |  |  |  |  |  |
| colon | 0.95 | 0.99 | 0.9822-0.9967 | 0.95 | 0.95 | 0.92 | 0.97 |
| liver | 0.87 | 0.99 | 0.9929-0.9993 | 0.85 | 0.98 | 0.99 | 0.87 |
| pancreas | 0.98 | 0.99 | 0.9934-0.9995 | 0.93 | 0.99 | 0.99 | 0.97 |
| Multiple organs | 0.99 | 0.99 | 0.9983-1.0000 | 0.96 | 0.99 | 0.98 | 0.99 |
| **Internal test set** |  |  |  |  |  |  |  |
| colon | 0.93 | 0.98 | 0.9663-0.9963 | 0.95 | 0.93 | 0.85 | 0.97 |
| liver | 0.98 | 0.99 | 0.9870-1.0000 | 0.93 | 0.98 | 0.88 | 0.99 |
| pancreas | 0.98 | 0.99 | 0.9708-1.0000 | 0.93 | 0.99 | 0.98 | 0.98 |
| Multiple organs | 0.95 | 0.98 | 0.9482-0.9975 | 0.80 | 0.99 | 0.97 | 0.95 |
| **External test set1** |  |  |  |  |  |  |  |
| colon | 0.97 | 0.94 | 0.8691-0.9941 | 0.83 | 0.98 | 0.69 | 0.99 |
| liver | 0.98 | 0.96 | 0.8852-0.9961 | 0.80 | 0.99 | 0.81 | 0.98 |
| pancreas | 0.98 | 0.98 | 0.9604-0.9964 | 0.76 | 0.99 | 0.72 | 0.99 |
| Multiple organs | 0.98 | 0.97 | 0.8965-0.9981 | 0.88 | 0.99 | 0.83 | 0.99 |
| **External test set2** |  |  |  |  |  |  |  |
| colon | 0.79 | 0.86 | 0.8112-0.9103 | 0.67 | 0.86 | 0.71 | 0.83 |
| liver | 0.86 | 0.79 | 0.6404-0.9049 | 0.74 | 0.92 | 0.60 | 0.86 |
| pancreas | 0.78 | 0.79 | 0.7-60-0.8580 | 0.72 | 0.92 | 0.60 | 0.81 |
| Multiple organs | 0.91 | 0.96 | 0.9216-0.9850 | 0.84 | 0.97 | 0.96 | 0.88 |
| **prospective set** |  |  |  |  |  |  |  |
| colon | 0.76 | 0.81 | 0.7309-0.8808 | 0.57 | 0.86 | 0.66 | 0.80 |
| liver | 0.79 | 0.89 | 0.8043-0.9652 | 0.85 | 0.78 | 0.43 | 0.97 |
| pancreas | 0.71 | 0.78 | 0.6571-0.8794 | 0.71 | 0.71 | 0.46 | 0.91 |
| Multiple organs | 0.96 | 0.77 | 0.6999-0.9722 | 0.60 | 0.98 | 0.99 | 0.96 |

a) Acc, accuracy; b)AUC, area under the receiver operating characteristic curve; c) CI, confidence interval; d) PPV, positive predictive value; e) NPV, negative predictive value.

**Supplementary Figure**
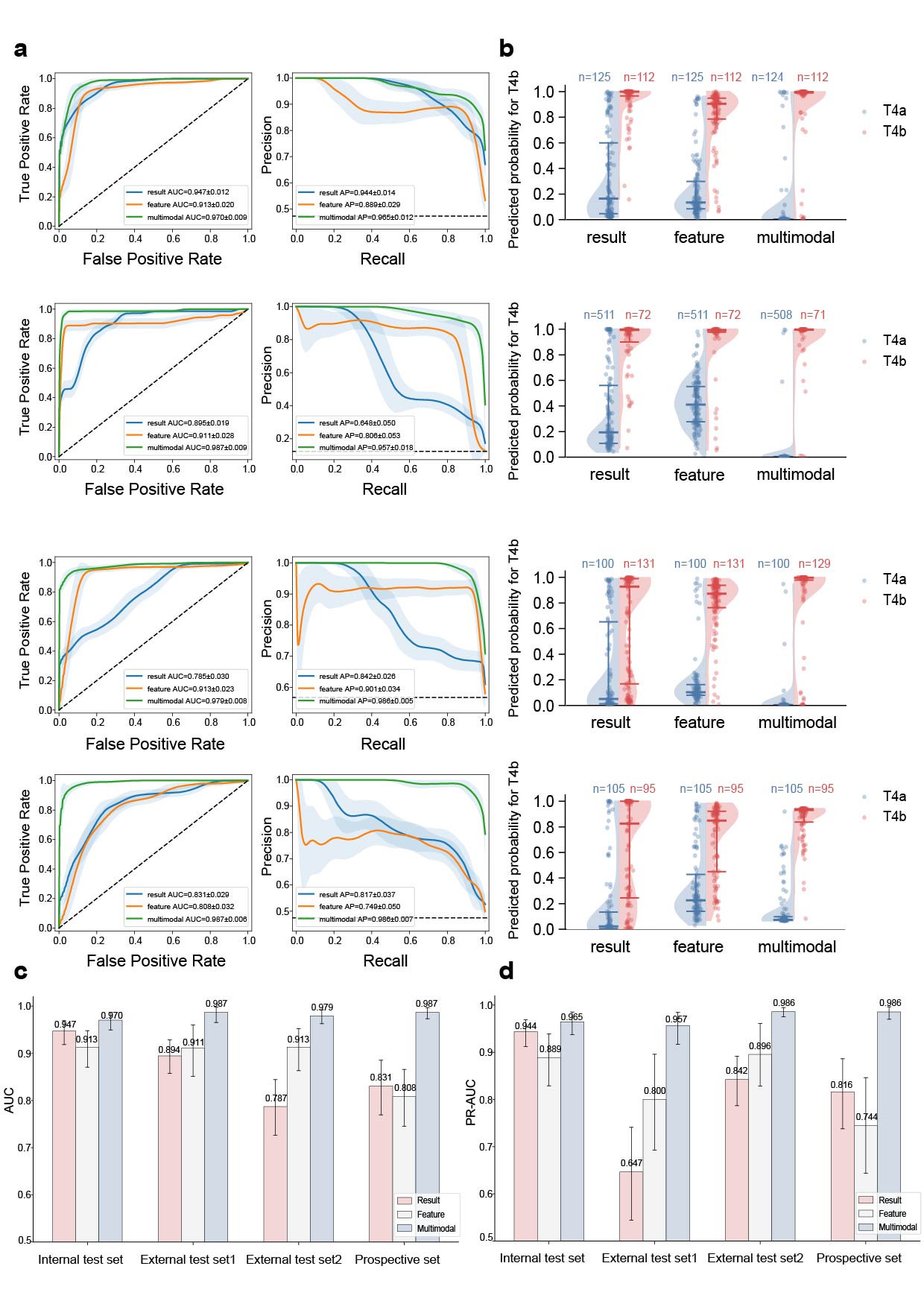


**Figure. S1. Ablation Study Evaluating the Effectiveness of the Transformer-based Fusion Framework**

(a) Receiver operating characteristic (ROC) curves and precision–recall (PR) curves of the three fusion strategies across the four independent test cohorts; (b) Violin plots illustrating the distribution of predicted probabilities for each fusion strategy. The blue regions represent the probability distributions for T4a cases, while the red regions represent those for T4b cases; (c–d) Bar plots summarizing the AUC and PR performance of the three fusion strategies across the four independent test cohorts.


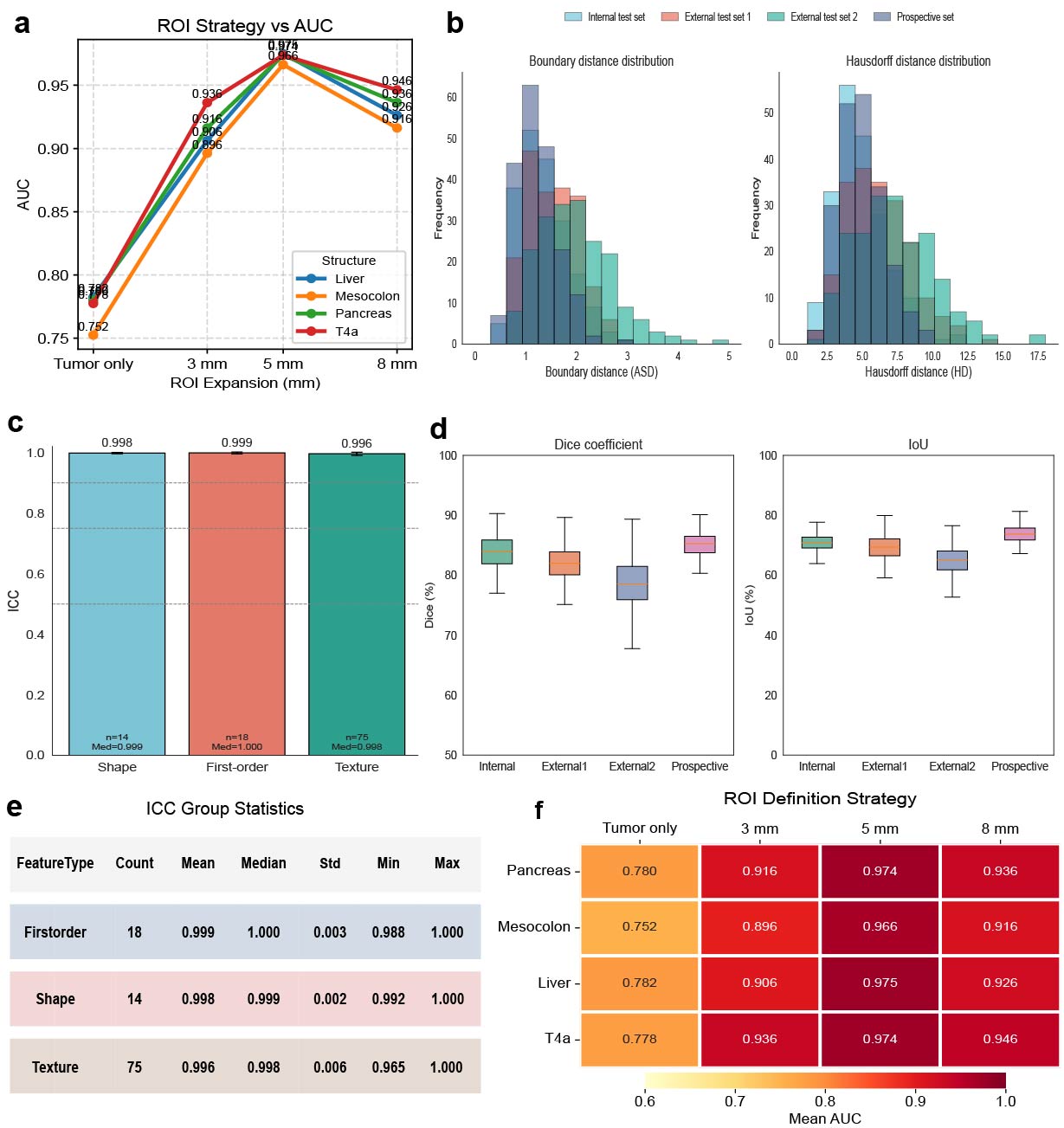


**Figure. S2. Impact of ROI Expansion and Multidimensional Feature Analysis on Model Performance**

(a) Line plots showing classification AUCs under different ROI expansion margins (3 mm, 5 mm, and 8 mm) for T4a and T4b cases across various adjacent organ invasion subtypes; (b) Histograms of boundary-based errors, including boundary distance and Hausdorff distance, summarizing the distribution of contour discrepancies; (c) Bar plots illustrating the relationship between radiomics features and the three-threshold consensus vector. Radiomics features are categorized into shape, first-order, and texture features; (d) Box-and-whisker plots showing Dice coefficient and Intersection over Union (IoU) across the internal validation cohort, external validation cohort 1, external validation cohort 2, and the prospective cohort. Boxes indicate the median and interquartile range (IQR), and whiskers represent the full range; (e) Summary table of radiomics features, including the number of shape, first-order, and texture features, along with their intraclass correlation coefficient (ICC) group statistics (corresponding to panel (c)); (f) Heatmaps of mean activation intensity under different ROI expansion margins (3 mm, 5 mm, and 8 mm) across various T4a and T4b organ invasion patterns, illustrating model response differences under diverse invasion scenarios.


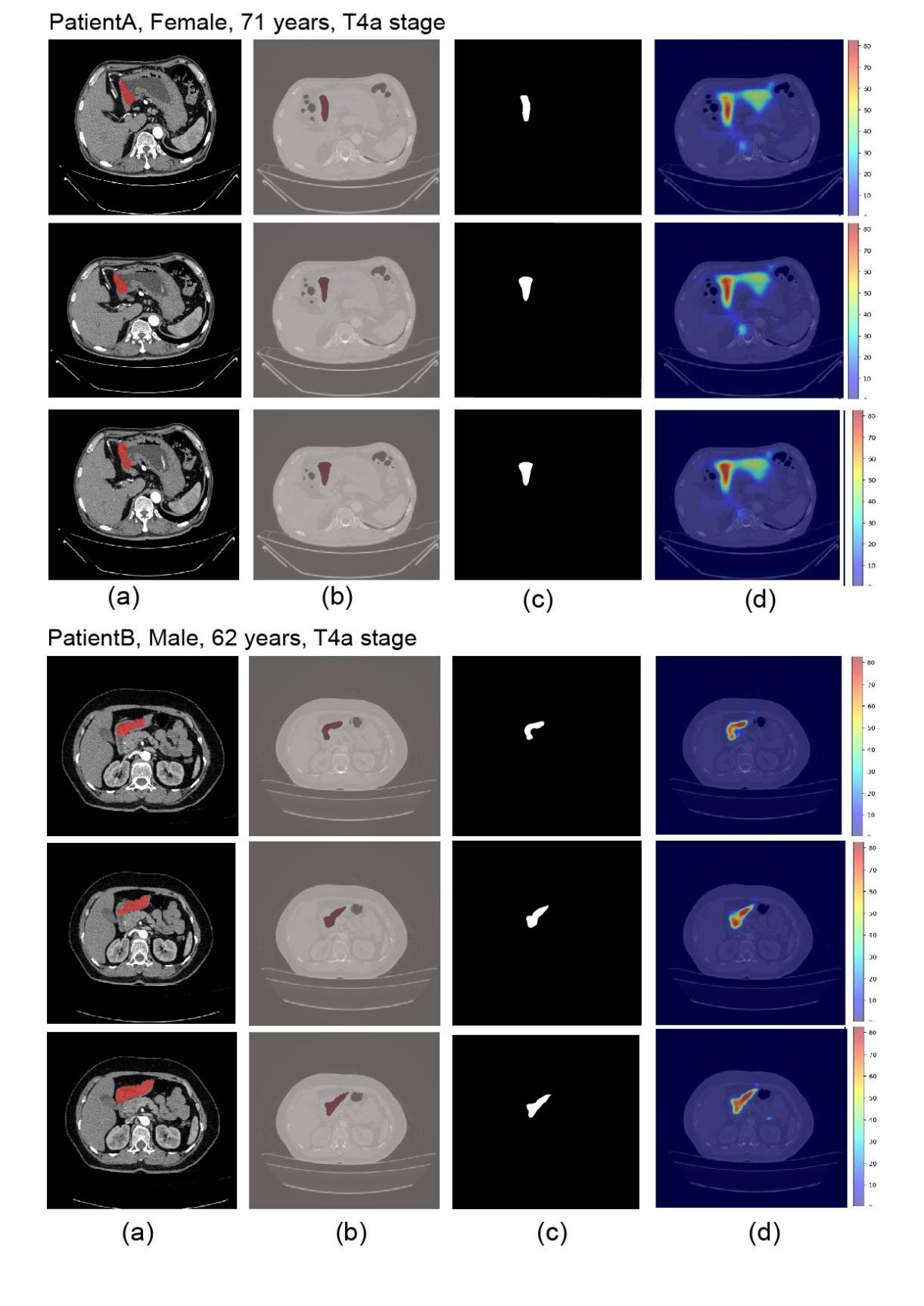


**Figure. S3. Segmentation Results Visualization**

Illustrative examples of gastric cancer lesion segmentation for two patients (a and b), showing three consecutive CT slices. The visualization includes: (a) original CT images overlaid with manual annotation masks; (b) U-Net prediction results overlaid on the CT images; (c) binary segmentation masks; (d) pixel-wise loss weight maps.


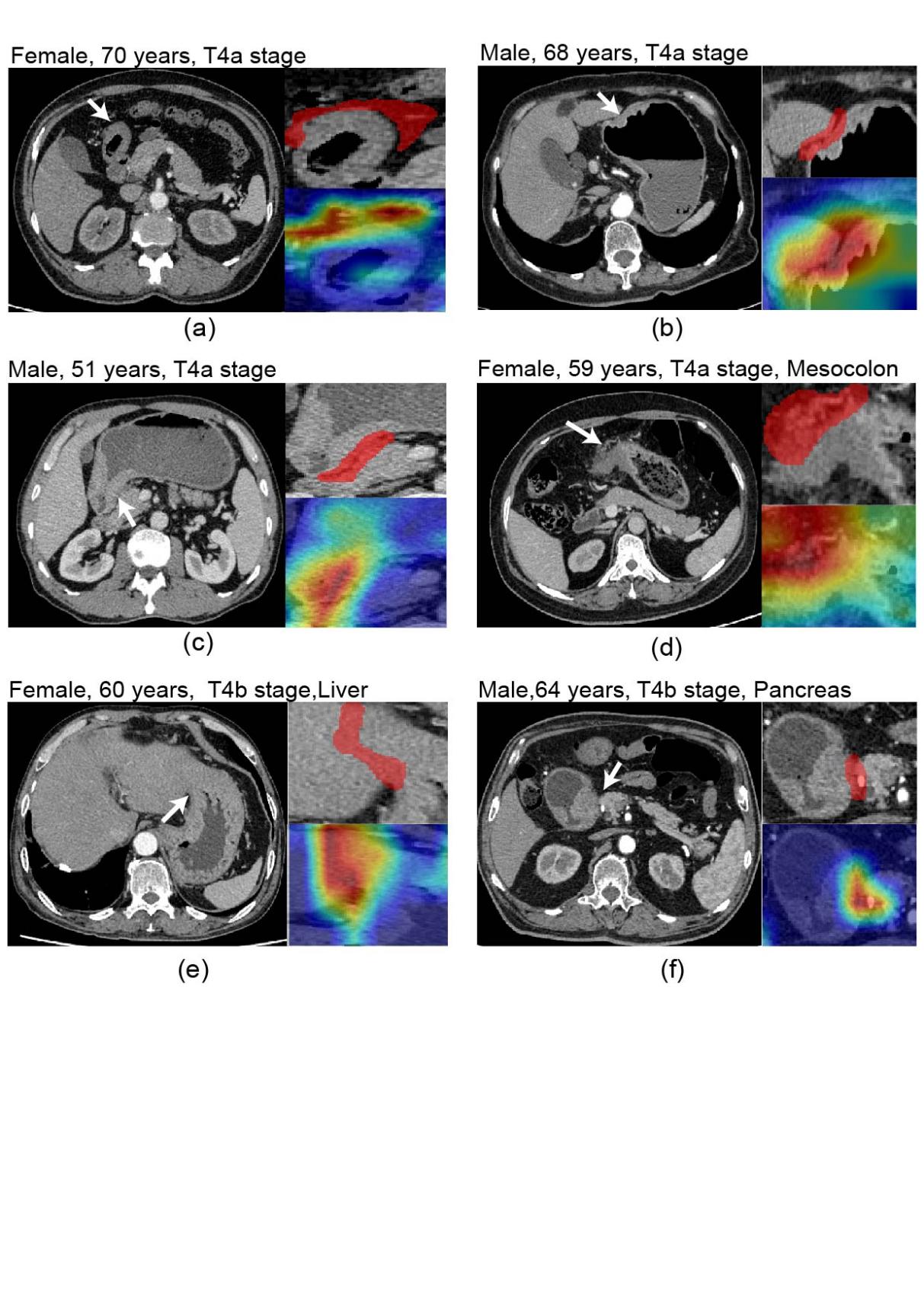


**Figure. S4. Grad-CAM Visualization of the Decision-Making Process of the GAVR Model**

(a–c) Representative T4a cases showing the original CT images and the corresponding Grad-CAM heatmaps; (d–f) Representative T4b cases with gastric cancer invading adjacent organs (colon, liver, and pancreas), along with the corresponding Grad-CAM heatmaps. In the Grad-CAM visualizations, red regions indicate areas of high model attention, whereas blue regions indicate low attention. Red bounding boxes highlight the key regions where the deep learning model focuses when identifying tumor invasion into adjacent structures in T4b cases. DL, deep learning; Grad-CAM, Gradient-weighted Class Activation Mapping; T4a, tumor invasion of the serosa; T4b, tumor invasion of adjacent structures.
